# The Impact of Oxytocin on Food Intake and Emotion Recognition in Patients with Eating Disorders: A Double Blind Single Dose Within-Subject Cross-Over Design

**DOI:** 10.1371/journal.pone.0137514

**Published:** 2015-09-24

**Authors:** Youl-Ri Kim, Jin-Sup Eom, Jae-Won Yang, Jiwon Kang, Janet Treasure

**Affiliations:** 1 Department of Neuropsychiatry, Seoul Paik Hospital, Inje University, Seoul, South Korea; 2 Institute of Eating Disorders and Mental Health, Inje University, Seoul, South Korea; 3 Department of Psychology, Chungbuk National University, Cheongju, South Korea; 4 Department of Psychology, The Catholic University of Korea, Bucheon, South Korea; 5 Section of Eating Disorders, Department of Psychological Medicine, King’s College London, Institute of Psychiatry, London, United Kingdom; University Hospital of Bellvitge-IDIBELL; CIBER Fisiopatología Obesidad y Nutrición (CIBERObn), Instituto Salud Carlos III; Department of Clinical Sciences, School of Medicine, University of Barcelona, Spain, SPAIN

## Abstract

**Background and Aim:**

Social difficulties and problems related to eating behaviour are common features of both anorexia nervosa (AN) and bulimia nervosa (BN). The aim of this study was to examine the impact of intranasal oxytocin on consummatory behaviour and emotional recognition in patients with AN and BN in comparison to healthy controls.

**Materials:**

A total of 102 women, including 35 patients with anorexia nervosa (AN), 34 patients with bulimia nervosa (BN), and 33 healthy university students of comparable age and intelligence, participated in a double-blind, single dose placebo-controlled cross-over study. A single dose of intranasal administration of oxytocin (40 IU) (or a placebo) was followed by an emotional recognition task and an apple juice drink. Food intake was then recorded for 24 hours post-test.

**Results:**

Oxytocin produced no significant change in appetite in the acute or 24 hours free living settings in healthy controls, whereas there was a decrease in calorie consumption over 24 hours in patients with BN. Oxytocin produced a small increase in emotion recognition sensitivity in healthy controls and in patients with BN, In patients with AN, oxytocin had no effect on emotion recognition sensitivity or on consummatory behaviour.

**Conclusions:**

The impact of oxytocin on appetite and social cognition varied between people with AN and BN. A single dose of intranasal oxytocin decreased caloric intake over 24 hours in people with BN. People with BN showed enhanced emotional sensitivity under oxytocin condition similar to healthy controls. Those effects of oxytocin were not found in patients with AN.

**Trial Registration:**

ClinicalTrials.gov KCT0000716

## Introduction

Oxytocin is now recognised as having a central role in the neural circuits involved in social behaviour, appetite, anxiety, and stress [1,2]. These features are also characteristic of people with an eating disorder, which raises the possibility that oxytocin is involved in the pathophysiology of the disorder [3,4]. During the starvation phase of anorexia nervosa (AN), the levels of oxytocin in the cerebrospinal fluid are decreased [5–7]. There are changes in the release of oxytocin in response to a meal in both the acute illness and post-recovery conditions [8]. Moreover, an intranasal adminstration of oxytocin produces changes in the attentional processing of cues that are salient for people with AN. For example, the vigilance toward food and body image stimuli is reduced [9]. In addition, the attentional bias toward negative facial emotions (disgust, anger) was modified by oxytocin in AN [10]. These findings suggest that oxytocin systems may be involved in fear-related stimuli and social cue processing in AN patients.

Meanwhile, fewer studies have investigated the oxytocin sytem in bulimia nervosa (BN) or in binge eating disorders although some of the features of these conditions also suggest a possible dysfunction in oxytocin systems. Oxytocin has been demonstrated to be an important peptide for body weight regulation [11,12]. Animal studies suggest that oxytocin is involved in weight control [13,14] and is particularly involved in inhibiting the appetite for sugar and carbohydrates [11,15]. Oxytocin receptor antagonist injections in wild-type mice produced a preference for sucrose over fat [16]. Oxytocin receptor knockout animals consume greater amounts of sweet solutions than wild types [17] and develop late onset obesity [18–20]. Animals engineered not to express oxytocin overconsume sweetened food [21] and carbohydrates [22]. Oxytocin expression was down regulated upon long-term intermittent exposure to sugar, which may represent a form of neural adaptation to a high sugar diet [23]. Mice with dietary-induced obesity exhibit functional abnormalities in the oxytocin systems. In humans, the administration of oxytocin in obese men produced weight loss [24]. Moreover, a recent study on healthy men found that oxytocin reduced the intake of high sugar snacks [25,26]. To our knowledge, there has been no study investigating oxytocin function in patients with BN or binge eating disorders.

In addition to playing a key role in appetite, oxytocin is involved in social and emotional processing. Problems that involve social emotional cognition may underpin some of the interpersonal difficulties in patients with eating disorders, such as a reduction in emotional expression in patients with AN and problems with trust and conflict in patients with BN, which are thought to contribute to the maintainance of the disorders [27]. Social cognition is complex and includes various subprocesses. A recent meta-analysis of the various components of social cognition in patients with eating disorders found an impairment in most of the domains of social functioning [28]. Emotional recognition was impaired in patients with eating disorders but with a large variation observed between tasks, and the weakest effects were observed for static photographs of faces. To our knowledge, a more ecologically valid technique to measure the sensitivity for emotional discrimination with dynamically changing facial expressions has yet to be used to examine eating disorders. In this study, we used this experimental paradigm to compare the emotional recognition sensitivity between people with AN or BN and healthy controls following a single dose of placebo or oxytocin.

The aim of this study was to examine the impact of an intranasal oxytocin challenge test on two possible maintaining factors of eating disorders: problems in appetite control and social cognition. The first hypothesis was that oxytocin would decrease food consumption. The second hypothesis was that social cognition would be improved in the oxytocin condition in patients with eating disorders.

## Materials and Methods

### Participants

The protocol for this study was approved by both the Institutional Review Board of the Korean Food and Drug Association (12061) and the Institutional Review Board of the Seoul Paik Hospital (IIT-2012-096) on September 16^th^, 2012 (See S1–S4 Texts). Although this is an experimental proof of concept study, and we have established with the regulatory agencies in the UK that it does not need to registered, we have found that other experts and journals find this confusing. Therefore, we have registered the study in the clinical trial registry [the Clinical Research Information Service (http://cris.nih.go.kr) (registration number: KCT0000716)]. All ongoing and related trials for this drug/intervention were registered. All of the participants provided written informed consent.

One hundred and two women (35 patients with AN, 34 patients with BN, and 33 healthy university students) completed this double-blind, placebo-controlled cross-over study. The first participant entered the study on April 4^th^, 2013, and the last participant completed follow-up on August 14^th^, 2014. The patients with AN were recruited from the inpatient ward and the patients with BN were recruited from the outpatient clinic of the Eating Disorders Clinic of the Seoul Paik Hospital in Seoul, South Korea. The diagnosis of eating disorder was confirmed by the Structured Clinical Interview from the Diagnostic and Statistical Manual of Mental Disorders, Fourth Edition [29]. All patients satisfied the DSM-5 diagnostic criteria of AN or BN. The exclusion criteria for comorbidities included active substance use disorder, psychotic disorder (schizophrenia, schizoaffective, psychosis not otherwise specified), and autism spectrum disorder. Patients taking psychiatric medications other than fluoxetine were also excluded.

The healthy controls were undergraduate or graduate students of a comparable age and intelligence who responded to an advertisement posted in the psychology department at a women’s university in Seoul, South Korea. The inclusion criteria were healthy females without a history of medical or psychiatric illness and a minimum age of 17. We screened all healthy participants for eating disorders with the SCOFF questionnaire [30]. The exclusion criteria for the healthy controls included a self-reported history of major depression, bipolar, panic, or psychotic disorders, substance dependence, epilepsy, eating disorder, autism spectrum disorder, traumatic brain injury, and taking medications (including contraceptives). We also excluded the participants who were smokers, homosexual, or parous because these conditions may interfere with oxytocin functioning.

The healthy controls and patients with BN who had their period were tested during the follicular phase of their menstrual cycle (approximately days 3 through 12). None of the patients with AN were menstruating. The participants were compensated for their travelling expenses and time.

The degree of the eating psychopathology for all participants (weight, shape, eating concerns, and dietary restraint) was evaluated using the Korean version of the Eating Disorder Examination self-report version Questionnaire (EDE-Q) [31]. Depression and anxiety were assessed for each subject by using the standardized Korean versions of the Beck Depression Inventory (BDI) [32] and the Spielberger State and Trait Anxiety Inventory (STAI) [33], respectively. The Korean version of the Ward 7-subtest short form of the Wechsler Adult Intelligence Scale [34] was used to measure the IQ of all participants.

**Fig 1:**
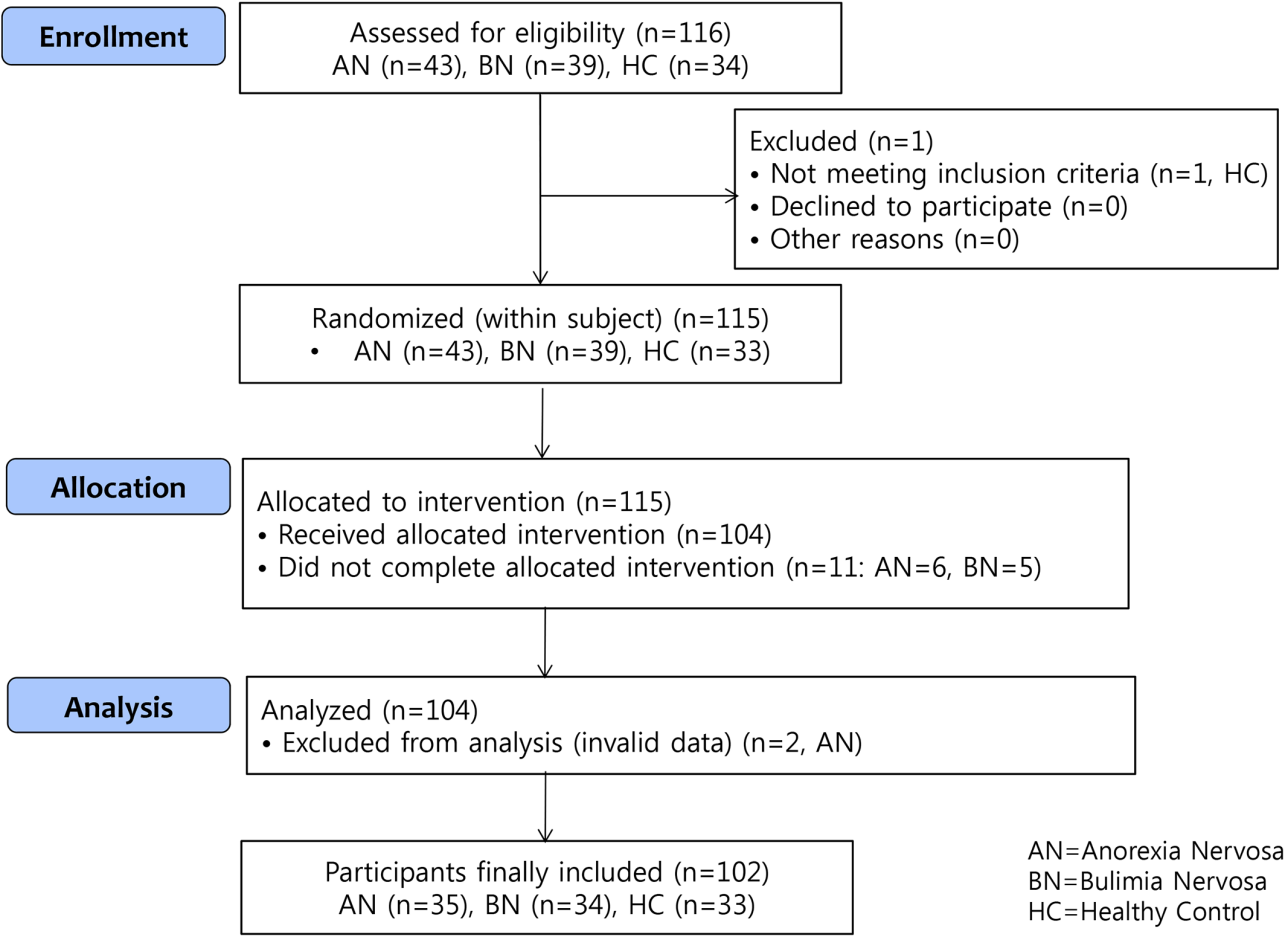
A consort diagram describing the participants in the study.

A consort diagram is shown in Fig 1 (See S5 Text).

### Oxytocin preparation

The intranasal oxytocin spray was formulated by *JW* Pharmaceuticals (Seoul, South Korea) from oxytocin powder (Hemmo Pharmaceuticals, Mumbai, India). The solution was prepared by combining 35.2 mg of oxytocin (568 U) with 300 mL of 0.9% sodium chloride solution and adjusting the pH to 4.01 with 10× diluted acetic acid. The placebo spray (pH 4.01) was formulated with 0.9% sodium chloride solution and acetic acid, without the addition of oxytocin. The filtered and sterilized solutions were sealed in individual vials (1.5 mL each) and were frozen for storage. On the day of use, the vials were thawed and kept in a refrigerator at 4°C until required. A clinician prepared the nasal spray by transferring the oxytocin or the placebo from the vial into a nebulizer. The nebulizer was primed and given to the participants, who self-administered the nasal spray while being monitored by the clinician.

### Dynamic facial morphing task

The dynamic facial morphing task consisted of video clips showing computer-morphed faces. Neutral faces morphed into emotional faces showing sad, fearful, angry, or happy expressions with 50 gradual levels of each emotion, adhering to the experimental paradigm described by Joormann and Gotlib [35]. The task was formulated by one of the authors [36] and was conducted using MATLAB version R2010 (Mathworks, Natick, MA, USA) in conjunction with the Psychophysics Toolbox version 2.54 [37,38]. The facial stimuli were (12.7 × 16.9 cm) in size and were presented in colour in the middle of the screen with a grey background. The animated morphs were presented on a high-resolution 15-inch monitor, and the distance between the participant and the monitor was of about 60 cm.

The participants were asked to respond as quickly as possible with a keyboard stroke when they noticed any emotion in the face. The stimuli included faces of two men and two women taken from a database of Korean actors and actresses in their 20s to 40s [39]. Four practice trials and 16 experimental trials, which comprised four emotions from each of the four chosen actors/actresses, were conducted. The outcome was measured as the intensity level at which emotions were accurately identified (; emotional sensitivity).

**Fig 2:**
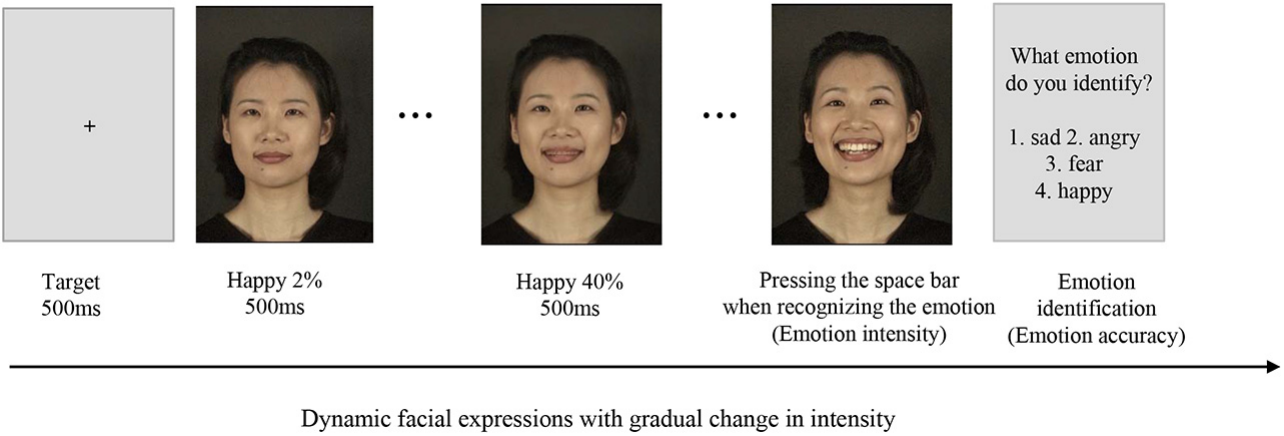
Dynamic facial morphing task. In the task, a fixed ‘+’ was initially shown for 500 ms in the centre of the screen, after which a neutral facial expression with 0% emotional intensity was shown for 500 ms. Progressive facial expressions of a 2% higher emotional intensity were shown at 500 ms intervals until the participant detected the presence of an emotion in the facial expression and reacted by pressing the space bar on the computer keyboard. The facial morphing was stopped, and the participant identified the emotion as ‘sadness’, ‘fear’, ‘anger’, or ‘happiness’ using the number keys ‘0’, ‘1’, ‘2’, or ‘3’, respectively, after which the next trial started immediately. The photograph is from a database published [39].

In the task, a fixed ‘+’ was initially shown for 500 ms in the centre of the screen, after which a neutral facial expression with 0% emotional intensity was shown for 500 ms. Progressive facial expressions of a 2% higher emotional intensity were shown at 500 ms intervals until the participant detected the presence of an emotion in the facial expression and reacted by pressing the space bar on the computer keyboard. The facial morphing was stopped, and the participant identified the emotion as ‘sadness’, ‘fear’, ‘anger’, or ‘happiness’ using the number keys ‘0’, ‘1’, ‘2’, or ‘3’, respectively, after which the next trial started immediately (Fig 2).

### Procedures

Each participant visited the laboratory twice for testing: once in the placebo condition and once in the oxytocin condition. Neither the participant nor the researchers involved were informed about the drug assignment of the day. The order in which to administer the placebo or the oxytocin to each participant was determined randomly (1:1) by the project coordinator, who was not involved in conducting the experiment, using Microsoft Excel as follows: (1) The RAND function was used to generate a random number between 0 and 1, (2) If the generated number was smaller than 0.5, the participant was given the placebo first and (3) If the generated number was greater than or equal to 0.5, the participant was given oxytocin first.

The patients with eating disorders were provided with meal plans with fixed-size portions during the experimental period in order to not influence the drug’s effect on calorie consumed during 24 hours. The patients with AN did not receive any direct support for eating during the 24 hours following the experiments on the inpatient ward. The patients with BN had meal plans focused on preventing binging and purging while supplying adequate energy to maintain a healthy weight, and they were instructed with a meal routine during the experimental period. Healthy controls were instructed to continue their routine diet during the 24 hours after the experiments.

The participants were tested in a private room at the Seoul Paik Hospital at 14:30. They were instructed to abstain from consuming alcohol or caffeine on the test day and to abstain from consuming food and drink (other than water) for 2 hours before the tests. Upon arrival, the participants completed baseline measures of physical symptoms, including abdominal, neurological, dermatological, and cardiac symptoms. The oxytocin and the placebo were self- administered intra-nasally under the supervision of a clinician, and the neuropsychological tasks were started 45 minutes later. A dose of 40 IU of oxytocin was chosen based on the results of a recent review that found the short-term use of intranasal oxytocin administered to male and female humans in dosages of up to 40 IU (per dose) to result in no detectable, subjective changes in a controlled research setting [40]. The nasal administration of oxytocin followed the guidelines established by Guastella et al. [41]. 90 min after the drug had been administered, the participants were asked to drink a carton of apple juice (Delmont, 190 mL per carton, 60 kcal). They were asked to consume as much of the juice as possible, as quickly as they were comfortable with, within a 20 min period. Finally, the participants completed an adverse symptom questionnaire. Over 24 hours following the experiment, the participants recorded a food diary to report every food and beverage items consumed, including the type, quantity, and approximate time and they sent back their diaries next day through their smartphones. A researcher who was blind to all the study procedures counted the calorie intake from the participant’s diary. The second appointment was scheduled at least four days and at most seven days after the first appointment. The same procedure was repeated during the next visit, but the content of the spray differed according to the randomization plan.

### Statistical analysis

Sample size was calculated to detect two-way interaction effect using a repeated-measures ANOVA based on mixed effect model with 95% power and correlation among repeated measure = 0.5 across 3 groups. The results showed that 22 subjects would be needed with a medium effect (*Δη* ^*2*^ = 0.06) and 129 subjects would be needed with a small effect (*Δη* ^*2*^ = 0.01) per each group to detect significant interaction effect.

At the placebo condition, one-way ANOVAs were calculated for the amount of juice drank, calories consumed during 24 hours, and sensitivity to emotional recognition to examine the baseline differences among the diagnostic subtype (AN, BN, controls), as we valued the findings at the baseline.

We explored a mixed factor analysis (ANOVA) defining the following factors: 1) pre-post measure for drug (intra-factor); 2) drug (oxytocin-placebo) (intra-factor); and 3) diagnosis (AN-BN-controls) (inter-factor). As the pre-post measure was not significant on outcome measures of juice intake (p = 0.825), calories consumed (p = 0.908) and emotional intensity (p = 0.859), we excluded carry-over effect of pre-post measure. Therefore, drug and diagnosis were included as factors in repeated measures ANOVA. For the food intake outcomes of the immediate juice intake and the calories consumed over 24 hours, we examined 2(drug) × 3(diagnosis) repeated-measures ANOVAs and then made an independent analysis stratified by diagnosis to cope with low power due to small sample size.

As the level of difficulty of the morphing task was low, the main dependent variable we used was emotional intensity not accuracy. The sensitivity of emotional recognition was restricted to trials in which facial expressions were correctly identified. The emotional intensity was converted into the corrected emotional intensity which was calculated from the relative emotional intensity at the point of identification of an emotion.

The responses to the emotional stimuli were investigated via two way 2 (drug) × 3 (diagnosis) repeated-measures analyses of variance (ANOVAs) on overall emotion, and then repeated-measures ANOVAs for each of the emotions (sad, fear, angry and happy) stratified by diagnosis were carried out. Post-hoc pairwise comparisons or simple main effect analyses were performed for significant main or interaction effects.

The data shown are presented as the mean and effect size (ES) if appropriate [Cohen’s *d* for paired *t*-tests; partial eta squared (*Δη* ^2^) for ANOVA]. Cohen’s *d* was described as negligible (< 0.20), small (< 0.50), medium (≥ 0.50 and < 0.8), or large (≥ 0.8) [42]. *Δη* ^2^ was described as small (<0.01), medium (<0.059), or large (<0.138) [43]. *P*-values <0.05 were considered to be significant, and two-tailed tests were used. The statistical analyses were performed using SPSS 19.0 (IBM Inc., Chicago, IL, USA).

## Results

### Demographic and clinical characteristics

The demographic and clinical characteristics of the participants are shown in Table 1. The age (p = 0.754) and IQ levels (p = 0.243) did not differ among the diagnoses. The mean age of onset (p = 0.957) and the mean duration of the illness (p = 0.243) did not differ between patients with AN and patients with BN. Patients with AN and BN had higher levels of depression and anxiety than controls [all p<0.001, BN = AN>HC in post hoc Tukey]. The eating disorder psychopathology differed among the diagnoses [all p<0.001: in post hoc Tukey, BN>AN>HC in global score and eating concern scale, BN>AN = HC in shape and weight concern scales, AN = BN>HC in restraint scale].

**Table 1:** Demographic and clinical characteristics of healthy controls and the patients with anorexia nervosa and bulimia nervosa.

Characteristics	AN (a, n = 35)	BN (b, n = 34)	HC (c, n = 33)	*F*(2,99)	*p*	Tukey HSD
Age, years	21.97(8.41)	23.03(5.17)	22.64(2.28)	0.283	0.754	NA
Age of onset, years	18.80(5.52)	18.74(4.38)	NA	*T(*_*67)*_ = -0.054	0.957	NA
Duration of illness, months	43.26(62.13)	57.59(34.72)	NA	*T(*_*67)*_ = 1.178	0.243	NA
WAIS, IQ	107.11(12.38)	106.62(11.91)	110.79(5.54)	1.581	0.211	NA
BMI	15.07(2.41)	20.24(2.45)	20.86(2.14)	63.334	<0.001	b = c>a
BDI	21.97(12.99)	18.29(10.94)	7.28(6.90)	16.788	<0.001	a = b>c
STAI						
State	55.85(13.62)	53.26(12.78)	43.44(11.27)	8.746	<0.001	a = b>c
Trait	54.94(13.29)	53.26(12.79)	43.75(11.16)	10.972	<0.001	a = b>c
EDE-Q						
Restraint	2.21(1.77)	2.32(1.78)	0.76(0.76)	10.927	<0.001	a = b>c
Eating Concern	1.88(1.69)	2.76(1.76)	0.56(0.77)	18.613	<0.001	b>a>c
Shape Concern	2.38(1.49)	3.56(1.82)	1.52(1.16)	15.343	<0.001	b>a = c
Weight Concern	2.73(1.45)	3.88(1.60)	2.21(1.29)	11.600	<0.001	b>a = c
Global	2.30(1.45)	3.13(1.48)	1.26(0.87)	17.413	<0.001	b>a>c

AN, anorexia nervosa; BN, bulimia nervosa; HC, healthy controls; WAIS: Wechsler Adult Intelligent Scale; IQ, Intelligent Quotient; BMI, body mass index; EDE-Q, Eating Disorder Examination Questionnaire; BDI, Beck Depression Inventory; STAI-State, Spielberger State and Trait Anxiety Inventory State score; STAI-Trait, Spielberger State and Trait Anxiety Inventory Trait score; NA, Not applicable

### Immediate consummatory behaviour of juice intake

Table 2 shows the results of immediate test meal of juice intake after receiving oxytocin or placebo. In the baseline condition, the amount of juice drank was significantly different among the groups [F(2,99) = 8.738, p<0.01; Δη^2^ = 0.150]. The post hoc tests showed that both the patients with AN [p<0.001] and BN [p = 0.030] drank a lower amount of juice than the controls did.

**Table 2:** The effect of oxytocin on immediate drinking juice and food intake for 24 hours in patients with anorexia nervosa and bulimia nervosa, and healthy university student.

	AN (n = 35)					BN (n = 34)					HC (n = 33)					*F*	*p*	*Δη* ^*2*^
	Placebo	Oxytocin	*t*	*p*	*Cohen’s d*	Placebo	Oxytocin	*t*	*p*	*Cohen’s d*	Placebo	Oxytocin	*t*	*p*	*Cohen’s d*			
Juice drunk (ml)	92.03(83.78)	97.49(81.97)	-0.537	0.595	-0.082	118.35(63.92)	131.50(61.19)	-1.662	0.106	-0.197	160.91(52.88)	172.82(43.31)	-1.924	0.063	-0.179	8.738	<0.001	0.150
Intake (kcal/day)	1988.55(729.79)	2151.52(873.33)	-1.492	0.145	-0.190	2757.84(1047.65)	2277.60(942.65)	2.528	0.016	0.560	2295.79(808.18)	2179.48(692.61)	0.882	0.384	0.136	6.386	0.002	0.115

AN, anorexia nervosa; BN, bulimia nervosa; HC, healthy controls.

Data are shown as mean (s.d.).

F: univariate ANOVA among 3 groups (AN, BN, HC) for placebo condition

t: paired t-test between placebo and oxytocin condition in each of the group

The two-way 2 (drug) × 3 (diagnosis) repeated measures ANOVA showed a small effect of oxytocin [F(1,99) = 4.469, p = 0.037, Δη^2^ = 0.043] and large effect of diagnosis [F(2,99) = 11.600, p<0.001, Δη^2^ = 0.190]. In the repeated measures ANOVAs stratified by diagnosis, there was no significant effect of oxytocin on juice intake in any diagnostic subtype.

### Caloric intake for 24-hours

Table 2 shows the caloric intake over a period of 24 hours after receiving oxytocin or placebo. In the baseline condition, the amount of calorie intake during 24 hours was different among the groups [F(2,99) = 6.581, p = 0.002, Δη^2^ = 0.120]. The post hoc tests showed that the patients with BN reported eating more calories than the patients with AN during the 24-hour period (p = 0.001 for AN vs BN; p = 0.372 for AN vs HC; p = 0.082 for BN vs HC).

The two-way 2 (drug) × 3 (diagnosis) repeated-measures ANOVA showed a significant interaction between diagnosis and drug [F(2,99) = 4.736, p = 0.011, Δη^2^ = 0.089], but the effects of each of the factors were weak [for diagnosis, F(2,99) = 3.099, p = 0.050, Δη^2^ = 0.060; for drug, F(1,99) = 2.835, p = 0.095, Δη^2^ = 0.028]. In the repeated measures ANOVAs stratified by diagnosis, there was a significant effect of drug in patients with BN [F(1,33) = 6.389, p = 0.016, Δη^2^ = 0.162]. The patients with BN consumed fewer calories after receiving oxytocin (p = 0.016, d = 0.560). There was no significant effect of oxytocin either in healthy controls (p = 0.384) or patients with AN (p = 0.145).

### Sensitivity of emotional recognition

The thresholds for the facial emotion identification in the oxytocin condition are depicted in Table 3. In the baseline condition, there was no effect of diagnosis on the threshold of emotional recognition [F(2,99) = 0.662, p = 0.518, Δη2 = 0.013]. There was an effect of emotional type [F(3,97) = 171.584, p<0.001, Δη^2^ = 0.844], in that happy emotion was easier to identify and angry emotion was less easy regardless of diagnostic subtype.

**Table 3:** Mean emotional intensity (%) of facial expressions made by patients with anorexia nervosa and bulimia nervosa, and by healthy controls.

Emotion	AN (n = 35)					BN (n = 34)					HC (n = 33)					*F*	*p*	*Δη* ^*2*^
	placebo	oxytocin	*t*	*p*	*Cohen’s d*	placebo	oxytocin	*t*	*p*	*Cohen’s d*	placebo	oxytocin	*t*	*p*	*Cohen’s d*			
Total	40.56(10.93)	40.85(10.11)	0.238	0.814	-0.032	42.06(8.83)	39.49(7.40)	2.021	0.052	0.285	42.58(8.22)	40.05(8.05)	2.128	0.041	0.280	0.662	0.518	0.013
Sad	41.438(11.285)	41.392(10.895)	0.037	0.971	0.005	44.259(12.729)	40.197(7.781)	2.261	0.031	0.399	44.253(8.504)	41.158(8.939)	2.407	0.022	0.304	1.289	0.280	0.026
Fear	41.467(10.455)	42.628(10.714)	-0.885	0.383	-0.127	42.907(7.143)	42.670(8.380)	0.175	0.862	0.026	44.218(8.813)	41.848(8.968)	1.380	0.177	0.258	1.155	0.319	0.023
Angry	49.227(13.727)	49.511(12.644)	-0.147	0.884	-0.026	52.433(9.130)	48.774(10.182)	1.647	0.109	0.332	51.864(9.756)	49.438(9.774)	1.517	0.139	0.220	0.988	0.376	0.020
Happy	30.121(12.474)	29.871(12.896)	0.128	0.899	0.023	28.657(12.288)	26.317(8.109)	1.351	0.187	0.211	29.994(10.697)	27.754(9.098)	1.656	0.107	0.202	0.146	0.864	0.003

AN, anorexia nervosa; BN, bulimia nervosa; HC, healthy controls.

Data are shown as mean (s.d.).

F: univariate ANOVA among 3 groups (AN, BN, HC) for placebo treatment

t: paired t-test between placebo and oxytocin treatment in each group

**Fig 3:**
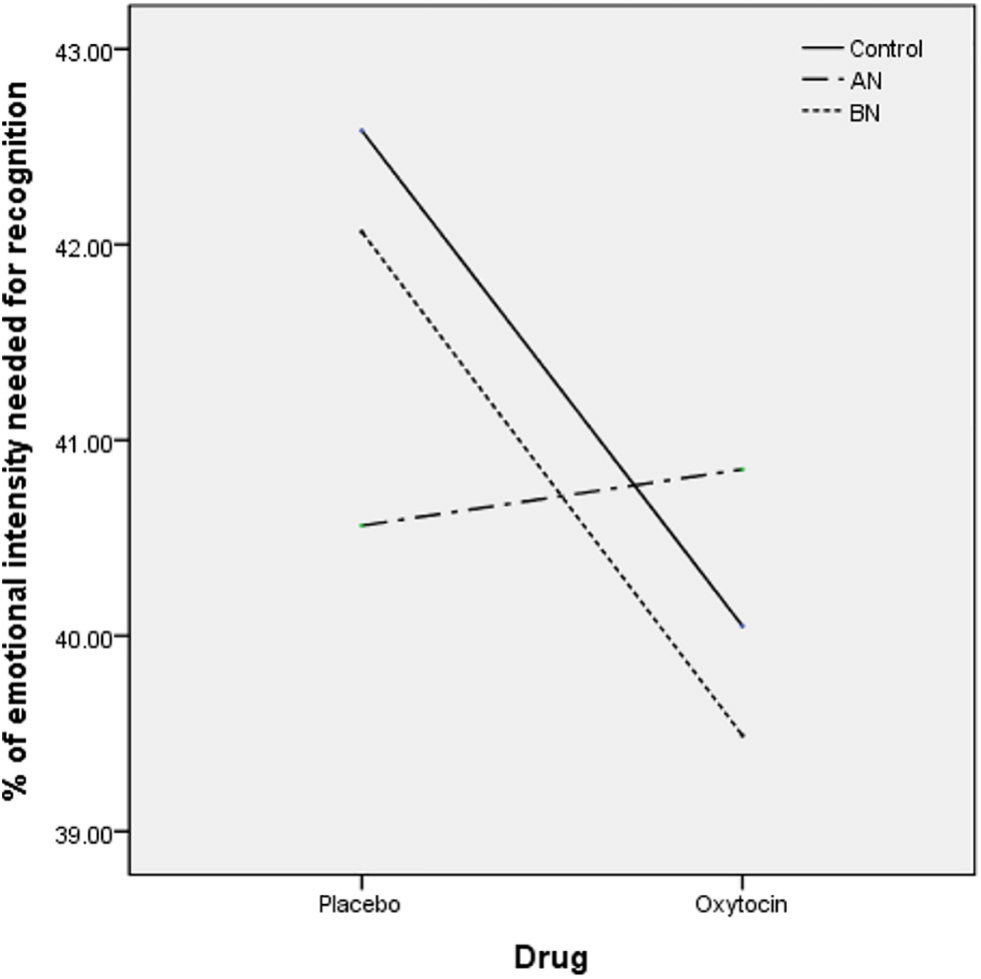
Emotional intensity for accurate emotion identification of total emotion under oxytocin/placebo conditions. Emotional intensity for accurate emotion identification of total emotion under oxytocin/placebo conditions. There was a main effect of drug [F(1,99) = 5.172, p = 0.025, Δη^2^ = 0.053], in which oxytocin increased the sensitivity of overall emotional recognition with a small effect size in the healthy women (d = 0.311) and the patients with BN (d = 0.315). AN, anorexia nervosa; BN, bulimia nervosa.

As the sample size was small, we made 2 (drug) × 3 (group) repeated-measures ANOVAs on overall emotion and then made an independent analysis stratified by diagnosis. The two-way 2 (drug) × 3 (group) repeated-measures ANOVAs demonstrated a main effect of drug [F(1,99) = 5.172, p = 0.025, Δη^2^ = 0.053] but no effect of group (p = 0.949) (Fig 3).

In the stratified analysis by diagnosis, there was a small effect of drug in overall emotional recognition [F(1,32) = 4.530, p = 0.041, Δη^2^ = 0.124] in healthy controls. In the post hoc analysis, the sensitivity to overall emotional recognition was increased in the oxytocin condition in the healthy controls [t(32) = 2.128, p = 0.041, d = 0.311]. In the following repeated-measures ANOVAs for each of the emotions in the healthy controls, the effect of oxytocin was significant in sad emotion [F(1,32) = 5.792, p = 0.022, Δη^2^ = 0.153]. In the post hoc analysis, the sensitivity to sad emotion recognition was increased in the oxytocin condition in the healthy controls (p = 0.022, d = 0.280).

In the patients with BN, there was a tendency of drug effect on overall emotional recognition [F(1,33) = 3.461, p = 0.072, Δη^2^ = 0.095]. In the post hoc analysis, oxytocin had a tendency to increase the sensitivity to overall emotional recognition in the patients with BN [t(32) = 2.128, p = 0.052, d = 0.315]. In the following repeated-measures ANOVAs for each of the emotions, the effect of oxytocin was significant in sad emotion in the patients with BN [F(1,33) = 5.144, p = 0.031, Δη^2^ = 0.135]. In the post hoc analysis, the sensitivity to sad emotion recognition was increased in the oxytocin condition in the patients with BN (p = 0.031, d = 0.399).

In the patients with AN, there was no effect of drug [F(1,34) = 0.348, p = 0.559, Δη^2^ = 0.010]. In the following repeated-measures ANOVAs for each of the emotions, there was no effect of oxytocin on the sensitivity to any of the emotion recognition in the patients with AN.

## Discussion

The aim of this study was to examine the impact of an intranasal oxytocin challenge test on two possible maintaining factors of eating disorders: problems in appetite control and social cognition. A single dose of intranasal oxytocin decreased caloric intake over 24 hours with a moderate effect in patients with BN. The immediate effect of oxytocin on intake was not significant in any of groups. Oxytocin increased emotion recognition particularly for sad expressions in patients with BN and in healthy women. No effects of oxytocin were found in either outcomes in patients with AN.

The most prominent finding in this study was that patients with BN consumed fewer calories over 24 hours after a single dose of intranasal oxytocin. The mechanisms underlying oxytocin’s reduction of caloric intake, particularly in the BN group, have not yet been defined. Previous work found that oxytocin reduced hedonic eating in test meal conditions in men [25, 26]. Possible mechanism underpinning this effect may be by reducing emotional eating through reducing stress [44]. A study of anti-obesity effects of oxytocin in free living conditions in obese rats [12] suggested that oxytocin may also affect energy homeostasis [11], glucose metabolism and lipid metabolism [12]. The positive results of this proof of principle experimental study suggest that it may be useful to further examine the role of oxytocin in patients with BN.

In addition to the impact on appetite and metabolism, oxytocin has an impact on social cognition. We used dynamic facial expressions to investigate emotion recognition as these resemble naturalistic interpersonal situations better than static facial expressions. We found that oxytocin improved emotion recognition for dynamic facial expressions, particularly for negative emotions in patients with BN and in healthy women. This result is consistent with a previous study [45] in which administering oxytocin enhanced overall emotion recognition of faces in healthy humans. Our results are also consistent with a previous study that used a similar emotion recognition tasks with morphing faces, in which oxytocin increased the sensitivity to emotion recognition in healthy men [46]. It is interesting that intranasal oxytocin did not change emotion recognition sensitivity in patients with AN even though they had a relatively higher dose per body weight of oxytocin due to their low weight. We previously reported an increase in methylation in the MT2 region of the OXTR promoter in patients with AN [47]. Therefore, it is possible that this reduced functionality accounts for the limited overall effects of oxytocin observed in this group.

There are some limitations in this study that need to be considered. First, we used a self-reported diary to record food consumption during the 24 hours observation period which may have decreased the sensitivity of the outcome measure. Objective measures of caloric intake may be more precise. Second, the technology used to examine the eating behaviour was simple. A design including a "taste test", a covert method of measuring the consumption of high fat, sugar palatable foods, would have been of value to test the hypothesis that oxytocin has a specific effect on hedonic eating. Similarly it would have been of value to measure the trait of food addiction. Third, three patients with AN and 3 patients with BN were taking stable dose of fluoxetine in our study, which may have confounded the results. It is common in clinical practice that patients with eating disorders take antidepressants which makes it ecologically relevant to include them in this sample. Furthermore, an additional analysis found no differences in the outcome measures in the response of this subgroup to the non-medicated patients. Fourth, it is possible that a more challenging social cognition task, such as “reading the mind” task, may increase the sensitivity to find differences. Lastly, other minor limitation was that the sample size was too small to detect a small interaction effect with a repeated-measures ANOVA. Therefore, it is possible that we could not detect any interaction effect on emotional intensity with this sample size.

In conclusion, the impact of oxytocin on appetite and social cognition varied between people with AN and BN. A single dose of intranasal oxytocin decreased caloric intake over 24 hours in people with BN. Moreover, people with BN showed enhanced emotional sensitivity under oxytocin condition similar to healthy controls. Those effects of oxytocin were not found in patients with AN.

## Supporting Information

S1 Dataset Raw data for participants. (SAV)

S1 Text Informed consent_English. (DOC)

S2 Text Informed consent_Korean. (DOC)

S3 Text Protocol_English. (DOC)

S4 Text Protocol_Korean. (DOC)

S5 Text Consort checklist. (DOC)
