# Supplementary material for: The Impact of Oxytocin on Food Intake and Emotion Recognition in Patients with Eating Disorders: A Double Blind Single Dose Within-Subject Cross-Over Design
Source: PLoS One. 2015 Sep 24;10(9):e0137514. doi: 10.1371/journal.pone.0137514 (PMC4581668; doi:10.1371/journal.pone.0137514)
Supplement: S1 Text — (DOC) [file pone.0137514.s002.doc]

**Information Sheet**

| Title : Oxytocin system as the mechanism of social emotional dysfunction in eating disorders  Institution : Department of Neuropsychiatry, Seoul Paik Hospital  researcher : Youl-Ri Kim, Eun-Young Jahang, Seung-Min Oh  Contact : 02-2270-0063, 02-2270-0970 |
| --- |

**1. Introduction**

- This research is to explore attentional bias after oxytocin application in the people who have eating disorder. It is intended for the people with eating disorder and the healthy people. For the deciding of whether you’ll participate in this research or not, you need to understand why and what this research is conducting. While you read this document, you can ask anything you want. Please make a deliberate decision.

**2. Purpose**

- The aim of this study is to investigate oxytocin system as the mechanism of social emotional dysfunction in eating disorders.

**3. Information about the participants and the drug using in the research.**

- This research is for the people with eating disorder and the healthy people. After injection of either oxytocin or placebo (normal saline), you’ll be assessed in terms of eating and affect. The research outcome will help us to figure out how anxiety about food and damage of social affective function develop in people with eating disorders.

- Oxytocin is an important neuromodulatory hormone which is in our human body. As a result of overall twenty years research analysis using intranasal oxytocin, it is proved oxytocin to be safe and have no side effects. The amount of oxytocin in this research, 40 IU, is not influential to the human body. Placebo doesn’t harmful in human body. We will conduct a neurocognitive assessment for detecting any change in eating symptom and emotion.

- The nasal spray you use will be disposed after single use. We don’t reuse it.

**4. Participants selection**

- We are inviting all women eating disorders who attend the Eating Disorders Clinic in Seoul Paik Hospital. Healthy women also are invited to participate in the study as a control group. This research uses random assignment, double blind test, cross-over design. You will use oxytocin and placebo in turn. Neither you nor researcher knows the order.

**5. Assessments and procedures**

- This research will be conducted through 2 times.

- In your first visit, you’ll get either one of oxytocin or placebo. After nasal application, you’ll conduct interview and questionnaire. Neuropsychological assessment will be started 45 minutes after the application of intranasal spray. It will take about 45 minutes to complete the neuropsychological tasks. After completing the task, you will be asked to drink apple juice as much as possible and be measured your height and weight. We will also collect your blood (2cc) and oral mucosa cell to examine any mutation of oxytocin gene. After come back home, you need to record your meal diary for 24 hours.

- You’ll visit after 1 week and conduct same procedure with first visit. The other reagent (oxytocin or placebo) which is different from the one of first visit will be applied. .

**6. Participant direction**

- You are not allowed to drink any alcohol or caffeine and food except water at least 2 hours before the experiments.

**7. Any experimental aspects of the study**

- Oxytocin has been considered to be an important neuromodulatory hormone that is influential in parenting, particularly maternal behavior. Also, oxytocin has been considered to be important in prosocial behavior between peers. A recent review of the influence of oxytocin nasal spray on social cognition concluded that overall oxytocin enhances prosocial behavior between peers. Also, it is proved that intranasal oxytocin is safe for the youth aged from 12 to 15 years.

**8. Risks**

- The amount of oxytocin using this research is not influential to the human body. It is reported that mild headache, dizziness, and chest tightness may occurr after IV injection, which are very rare in nasal spray. The effect of intranasal oxytocin completely disappears within 4 hours.

**9. Benefits**

- The benefits of oxytocin are not proved yet. You also may not have direct benefit. Once you have an application of oxytocin as participants, there is a possibility of improvement in eating symptom, affective experience, and anxiety. This may lead to the better condition. If you want, you can get the information about the result of assessment after the study completed.

- You can contribute to the development of therapy of eating disorder.The findings of this study will be helpful for the people who have eating disorders to be cared in future.

**10. Other treatment**

- We will offer continuous medical care for you regardless of being participant in the study.

**11. Reimburshments**

- You don’t need to pay any extra charge for this participation. Highly valued oxytocin and computerized nuerocognitive assessment are provided for free. After finishing all the procedures, you will be given 20,000 Korean won in case you are patients or 40,000 Korean won in case you are healthy women.

**12. Compensation for the risk**

- Side effects of oxytocin rarely happen. The clinician (Principal investigator YK) will take care of these symptoms immediately in Seoul Paik Hospital, and you don’t need to charge for this. If you involve in any other risk, we will instantly notify it to Institutional Review Board of Seoul Paik Hospital.

**13. Expecting participants**

- We are expecting that there are about 50 people with eating disorder and 50 healthy women to participate in this research. This research is supported by Korean National Research Foundation.

**14. Right to Refuse or Withdraw**

- You do not have to take part in this research if you do not wish to do so. You may also withdraw participation in the research at any time you choose. It is your choice and all of your rights will still be respected

**15. Additional information**

- In case of getting more information that can affect on your willingness to participate this research consistently, we will inform you immediately.

**16. Confidentiality**

- The information that we collect from this research project will be kept confidential. Information about you that will be collected during the research will be put away and no-one but the researchers will be able to see it. Any information about you will have a number on it instead of your name. Only the researchers will know what your number is and we will lock that information up with a lock and key. It will not be shared with or given to anyone except us.

**17. Whom to contact**

- You can get additional information during the research. If you have any questions about the research, please call this number below. You also can contact with Institutional Review Board of Seoul Paik Hospital for the request.

- Principal Investigator: Professor Youl-Ri Kim 02)2270-0970

- Researcher: Eun-Young Jahang / Seung-Min Oh

02)2270-0063, 02)2270-0557

- Institutional Review Board of Seoul Paik Hospital : 02)2270-0945

**Informed Consent**

| Title : Oxytocin system as the mechanism of social emotional dysfunction in eating disorders  Institution : Department of Neuropsychiatry, Seoul Paik Hospital  researcher : Youl-Ri Kim, Eun-Young Jahang, Seung-Min Oh  Contact : 02-2270-0063, 02-2270-0970Please initial box  1. I confirm that I have read and understand the information sheet. |
| --- |
| 2. I understand that my consent is entirely voluntary and that I may withdraw at any time, without giving any reason, without my medical care being affected. |
| 3. I authorize responsible individuals from the Trust and sponsor, Seoul Paik Hospital, etc to access my records or data. |
| 4. I agree to take part in the above study. |

____________________     ______________________    ______________________

 Your name                           Signature  Date

____________________     ______________________    ______________________

 Investigator                          Signature  Date

____________________     ______________________    ______________________

 Principal Investigator         Signature  Date

| Consent for  Genetic Testing and Research | | | | | Document No. | |
| --- | --- | --- | --- | --- | --- | --- |
|  |  |  |  |  |  |  |
| Test Subject | N a m e |  | A d d r e s s |  | | |
|  | Phone No. |  | Date of birth |  | | M/F |
| Legal guardian | N a m e |  | Phone No. |  | | |
| Genetic Testing/Research  Institution | N a m e | Seoul Paik Hospital | Phone No. | 02-2270-0063 | | |
| 1. The purpose of this genetic test or research? Oxytocin system as the mechanism of social emotional dysfunction in eating disorders   2. The specimen which has been used for the genetic test or research  □ will be disposed of immediately after the completion of the test or research.  □ will be stored after the completion of the test or research. (□ five years or years)  ※ The specimens which have been provided to the Gene Bank or Genetic Research Institutions will be stored in accordance with the rules of each institution.  3. The specimens remaining after the genetic test or research will be provided to the  Gene Bank or Genetic Research Institutions and made available for research projects conducted to prevent or treat diseases. Do you agree? □ Yes. □ No.  3-1 By ticking "Yes", the following information can be included when the specimens are provided;  □ both personal and clinical-epidemiological information.  □ only clinical-epidemiological information.  □ none of them.  ※ Personal information means personally identifiable information such as names.  ※ It is recommended that you get a full explanation from the counsellor about the following statements.  1) Specimens whose storage period has expired will be disposed of in compliance with the procedure  and method described in article 13 of Waste Control Act. And when specimens cannot be stored  due to closure of Genetic Testing Institutions or other unavoidable reasons, they will be transferred as stipulated in the Act.  2) Even if you have given consent, you may at any time withdraw the consent before the research starts. And genetic test results (except research results) will be preserved for ten years and when you or your legal guardian request, you or your legal guardian may access to genetic test results,  genetic testing and research consent, the record of provision of specimens to the Gene Bank or  Genetic Research Institutions.  3) This institution will undertake necessary measures to protect your personal information, and all research will be conducted only after review and approval by the Institutional Bioethics Committee.  4) You are not entitled to products such as medicines or diagnostic instruments, or patents based on  results from research projects using your specimens. And the results of research using your specimens and offered information will be presented at academic conferences or published in academic journals under the names of researchers and your information will not be exposed.  I confirm that I have been fully informed of this genetic test and research and have voluntarily consented to the above test or study. | | | | | | |
| Signature of test subject : date :  Signature of legal guardian : date :  Signature of counselor : date : | | | | | | |
| ※ Legal guardians should submit a document proving their status. | | | | | | |
