# Supplementary material for: The Impact of Oxytocin on Food Intake and Emotion Recognition in Patients with Eating Disorders: A Double Blind Single Dose Within-Subject Cross-Over Design
Source: PLoS One. 2015 Sep 24;10(9):e0137514. doi: 10.1371/journal.pone.0137514 (PMC4581668; doi:10.1371/journal.pone.0137514)
Supplement: S2 Text — (DOC) [file pone.0137514.s003.doc]

**연구과제 피험자 설명서**

| 연구제목 : 섭식장애 유지모델로서 사회 정서기능의 이상 및 옥시토신 메카니즘의 이상 규명  수행기관 : 서울백병원 정신건강의학과 섭식장애클리닉  연구자 : 김율리, 장은영, 오승민  연락처 : 02-2270-0063, 02-2270-0970 |
| --- |

**1. 개요**

- 이 연구는 섭식장애를 앓고 있는 환우들의 회복을 돕고자 섭식장애의 병태생리를 규명하고 효과적인 치료방법을 개발하기 위한 연구입니다. 본 연구는 섭식장애를 앓고 계신 분들과 정상 대조군을 대상으로 합니다. 귀하가 본 연구에 참여할 것인지 여부를 결정하기 전에, 본 연구가 왜 수행되고, 무엇을 수행하는지 이해하는 것이 필요합니다. 이 문서를 읽으면서 어떤 질문이라도 할 수 있으며, 숙고한 후 결정하십시오.

**2. 연구목적과 배경**

- 본 연구의 목적은 섭식장애에서 음식에 대한 불안과 사회정서 기능의 손상을 규명하기 위함입니다.

**3. 참여대상 및 연구에 사용되는 시약에 대한 정보**

- 본 연구는 섭식장애를 앓고 있는 분 및 정상인 분을 대상으로 하며, 옥시토신이나 위약을 투여한 후 섭식 및 정서 상태에 대해 평가하게 됩니다. 연구결과 옥시토신 귀하의 음식에 대한 불안 및 사회정서 기능 손상을 얼마나 개선할 수 있는지를 파악할 수 있습니다.

- 옥시토신은 인체에서 생성되어 인간의 체내에 존재하는 호르몬입니다. 옥시토신 비강 내 투여를 이용해 전세계에서 20년 동안 수행된 모든 연구를 분석한 결과 옥시토신 비강 내 투여는 안전하고, 부작용이 없는 것으로 나타났습니다. 따라서 본 연구에서는 섭식증상 및 정서에 있어서의 미세한 개선을 신경인지검사를 통해 평가하고자 합니다.

- 본 연구에서 귀하가 사용하는 비강스프레이는 1회 사용 후 폐기하며 재사용하지 않습니다.

**4. 시험군 또는 대조군에 배정될 확률**

- 본 연구는 무작위배정, 이중맹검, 크로스오버(cross-over)방식으로 진행됩니다.

즉, 귀하는 옥시토신 과 위약을 교대로 사용하며, 그 순서는 귀하 및 연구자가 모두 알지 못합니다.

**5. 본 연구에 참여하게 됨으로써 받게 되는 검사 및 절차**

- 본 연구는 2회에 걸쳐 수행됩니다.

- 첫 방문에서, 귀하는 옥시토신과 위약 중 하나를 투여 받습니다. 투여 후 대기시간 동안 질환 상태를 평가하기 위한 면담과 설문지를 수행하십니다. 신경심리검사는 스프레이를 사용한 45분 후에 시작할 것이며 이 검사를 통해 변화를 평가하십니다. 실험을 마친 후 키와 몸무게 측정 및 원하는 만큼의 음료수 섭취 후 섭취량을 측정합니다. 귀가 후 1일간의 식사일기를 기록합니다. 한편 귀하의 옥시토신 유전자의 다형성을 파악하기 위해 채혈(2cc) 및 면봉을 이용한 구강점막세포 채취를 합니다.

- 두 번째 방문은 1주일 이내에 시행하며, 귀하는 첫날 투여 받은 것과는 다른 시약(옥시토신 혹은 위약)을 투여 받은 후 동일한 절차를 수행합니다. 위약은 증류수와 용매만으로 되어 있으며 인체에 영향을 주지 않습니다. 실험을 마친 후 귀하의 성격상태를 평가하는 면담을 수행하십니다.

**6. 피험자가 준수해야 할 사항**

- 연구 당일에는 알코올과 카페인 섭취를 금합니다. 시약을 투여 받기 2시간 전에는 음식과 물을 제외한 음료 섭취를 하지 않습니다.

**7. 검증되지 않은 실험적인 측면**

- 옥시토신은 인체 내 자연적으로 생성되는 안전한 호르몬이며, 의학적으로는 태아안전성 분류 A등급으로 임산부에게도 무해한 성분입니다. 옥시토신은 오래 전부터 정맥주사 방법으로 산모 및 수유부에서 사용되고 있습니다. 옥시토신 정맥주사 후 경한 두통이나 구역감 등의 부작용이 드물게 보고된 적이 있었으나 수시간 내 소멸되며 그 이상의 부작용은 없었습니다. 비강 스프레이는 정맥투여에 비해서도 안전한 방법입니다. 성인의 경우 옥시토신 투여의 안전성은 입증되었습니다.

**8. 본 연구에 참여함으로써 예견되는 위험 또는 불편에 관한 사항**

- 본 연구에서 사용되는 옥시토신은 인체에 주관적인 변화를 일으키지 않는 정도의 양입니다. 매우 드물지만 옥시토신 정맥투여시 경한 부작용 (예, 경한 두통 및 경한 어지럼증, 약간의 답답함)을 경험했다는 보고가 있으나 비강투여의 경우 그 가능성은 희박합니다. 옥시토신 비강 내 투여의 직접적인 효과는 4시간 내에 완전히 소멸됩니다. 한편 귀하는 채혈절차에서 다소 따끔한 고통을 느낄 수도 있으며 점막세포 채취를 위해 구강을 면봉으로 문지르는 상황이 다소 불편을 초래할 수 있습니다. 귀하가 의학적으로 주의가 필요한 어떤 증상을 경험했을 경우 연구상황에서 주치의사가 귀하의 불편함에 대한 파악과 의문사항에 대해 적극 대처할 것입니다.

**9. 본 연구에 참여함으로써 기대되는 이익**

- 귀하가 본 연구에 참여함으로써 얻을 수 있는 치료에 대한 이익은 본 연구에 참여하지 않았을 경우에 얻는 이익과 동일합니다. 연구의 일환으로 옥시토신 투여를 받게 되면, 섭식 증상, 대인관계 및 불안의 개선을 경험할 수 있으며 이는 향후 섭식장애의 치료에 도움이 될 수 있습니다.

- 귀하가 원하신다면 본 연구가 모두 종료된 후 귀하의 평가결과를 통보 받으실 수 있습니다. 나아가서는 본 연구에 참여함으로 향후 섭식장애 환자들이 보다 나은 치료를 받는데 큰 도움이 될 수 있습니다.

- 옥시토신의 섭식장애에 대한 이익은 현재로서는 완전히 확증된 단계는 아니며, 1회성 처치로는 귀하가 본 연구에서 직접적인 이익을 얻지 못할 수도 있습니다. 그러나 귀하가 참여함으로 향후 다른 섭식장애 환자들이 보다 나은 치료를 받는데 도움이 될 수 있습니다.

**10. 섭식장애의 다른 치료법**

- 서울백병원 섭식장애 클리닉은 본 연구에의 참여여부와는 무관하게 귀하의 섭식장애 회복을 위한 표준의 치료방법들을 지속할 것입니다.

**11. 연구에 참여함으로써 지불해야 할 비용**

- 본 연구에 참여함으로써 귀하가 지불할 별도의 비용은 없습니다. 연구의 절차로 시행하게 될 고가의 옥시토신과 컴퓨터화된 신경인지평가 등은 모두 무료로 시행됩니다. 연구절차를 모두 마치면 환자의 경우2만원, 대조군의 경우 4만원이 한국연구재단에서 귀하의 계좌로 입금됩니다.

**12. 연구도중 발생한 피해에 대한 보상**

- 옥시토신 투여로 인한 직접적인 부작용들은 매우 드물지만 쇽, 과민증 등이 있습니다. 이러한 증상들은 본 연구책임자가 즉각적으로 의학적 도움을 구하여 처치할 것이며 이러한 부작용을 응급 처치하는데 발생되는 직접적인 의료비용은 연구자가 부담합니다. 그 외의 추가적인 보상은 없습니다. 다른 위험이 발생할 경우 의료진은 즉각 그 사실을 주지하여 서울백병원 임상시험심사위원회에 알리겠습니다.

**13. 예상 참여기간 및 본 연구에 참여하는 대략의 전체 피험자 수**

- 본 연구에는 섭식장애로 진단받은 약 50명의 참가자 및 동수의 건강한 정상인이 이 참여할 것으로 예상합니다. 본 연구는 어떤 회사로부터도 상업적인 후원을 받지 않는 순수한 연구목적으로 수행됩니다. 본 연구는 대한민국 교육과학기술부의 지원을 받고 있습니다.

**14. 연구에 자발적 참여 및 참여중단**

- 이 연구에 대한 참여는 전적으로 귀하의 선택입니다. 따라서 연구 참여를 원치 않을 경우라도 이 병원에서 치료를 받는데 불이익을 받거나, 다른 환자와 차별 받는 일 없이 치료를 지속할 수 있습니다. 또한 귀하가 연구에의 참여를 결정한 후라도 귀하는 이유를 대지 않고 언제든지 참여 동의를 철회할 수 있고 철회 시점 이후의 연구 참여를 중단할 수 있습니다.

**15. 연구 참여 도중 새로운 정보의 제공**

- 이 연구에 지속적으로 참여할 귀하의 의지에 영향을 줄 수 있는 새로운 정보가 수집되면 적시에 귀하에게 알려 드릴 것입니다.

**16. 피험자의 신원에 대한 비밀보장**

- 귀하의 신원을 파악할 수 있는 기록은 비밀로 유지할 것이며, 본 연구의 결과가 출판될 경우에도 귀하의 이름은 공개되지 않을 것이며 이니셜과 피험자번호에 의해서만 확인할 수 있습니다. 하지만, 본 연구의 점검을 실시하는 자, 임상시험심사위원회 및 관련 정부기관장은 피험자의 비밀보장을 침해하지 않고 관련규정이 정하는 범위 안에서 연구의 실시절차와 자료의 신뢰성을 검증하기 위해 본인의 의무기록을 직접 열람할 수 있습니다. 본 동의서에 서명함은 이러한 자료의 직접 열람을 허용한다는 것을 의미하며, 연구의 결과가 출판될 경우 피험자의 신원은 비밀 상태로 유지될 것입니다.

**17. 문의처**

- 귀하는 본 연구기간 동안에 연구자에게 언제든지 추가적인 정보를 요청할 수 있으며 이와 관련하여 질문이 있으시면 아래의 번호로 연락하시기 바랍니다. 또한 귀하는 피험자로서의 귀하의 권리에 대해 의문이 있을 경우 임상시험심사위원회로 연락할 수 있습니다.

- 연구책임자 : 정신건강의학과 김율리 02)2270-0970

- 연구담당자 : 정신건강의학과 장은영/오승민

02)2270-0063, 02)2270-0557

- 임상시험심사위원회 : 02)2270-0945

**피험자 동의서**

1. 본인은 본 연구에 대해 설명을 듣고 상기 피험자 설명문을 읽고 이해하였습니다.

2. 본인은 연구에 대한 모든 질문에 대한 답변을 듣고 결정을 내릴 충분한 시간을 얻었습니다.

3. 본인은 이 연구에 참여하는 것에 자발적으로 동의합니다.

4. 본인은 이후에 치료에 영향을 받지 않고 언제든지 연구의 참여를 거부하거나 연구의 참여를 중도 철회할 수 있고 이러한 결정이 나에게 어떠한 해가 되지 않을 것이라는 것을 알고 있습니다.

5. 본인은 이 동의서에 서명함으로써 연구목적의 나의 정보가 관련법이나 규정에 의해 허용되는 범위 안에서 연구자가 수집하고 처리하는데 동의합니다.

6. 본인은 이 동의서의 사본을 제공받을 것임을 알고 있습니다.

년 월 일

피험자 성명 서 명 서명일

년 월 일

법정대리인 성명 서 명 서명일

(해당되는 경우)

년 월 일

입회인 성명 서 명 서명일

(해당되는 경우)

년 월 일

연구자 성명 서 명 서명일

| [별지 제20호서식] <개정 2009.12.31> | | | | | | |
| --- | --- | --- | --- | --- | --- | --- |
| **유전자 검사·연구 동의서** | | | | | 동의서 관리번호 | |
|  |  |  |  |  |  |  |
| 검사·연구대상자 | 성명 |  | 주소 |  | | |
|  | 전화번호 |  | 생년월일 |  | | 남/녀 |
| 법정대리인 | 성명 |  | 전화번호 |  | | |
| 유전자검사·연구기관 | 기관명 | 서울백병원 | 전화번호 | 02-2270-0063 | | |
| 1. 유전자검사·연구의 목적: 섭식장애 병인규명을 위한 유전학적 연구  2. 위의 검사·연구 목적으로 기 사용한 검사대상물의 처리: □ 검사·연구 후 즉시 폐기  □ 검사·연구 종료 후 보존 (□ 5년 □ 년)  **※ 유전자은행·유전자연구기관에 제공된 검사·연구대상물은 해당 목적에 따라 보존됩니다.**  3. 위의 검사·연구목적으로 사용 후 남은 검사대상물을 유전자은행·유전자연구기관 등에 제공하여 질병의 예방·치료법의 개발 등에 활용하는 것에 대한 동의 여부: □ 동의함 □ 동의하지 않음  3-1 동의 시 귀하의 개인정보 또는 임상·역학정보 포함여부: □ 개인정보 및 임상·역학정보 포함 □ 임상·역학정보만 포함 □ 모두 동의하지 않음  **※ 개인정보란 성명 등 특정개인을 식별할 수 있는 정보를 말합니다.**  ※ 다음 각 항목에 대해서는 상담자로부터 충분한 설명을 듣도록 하십시오.  1) 보존기간이 경과한 검사대상물은 「폐기물관리법」 제13조에 따른 기준과 방법에 따라 폐기되며, 유전자검사기관의 폐업, 그 밖의 부득이한 사정으로 검사대상물을 보존할 수 없는 경우에는 법에서 정한 절차에 따라 검사대상물을 이관합니다.  2) 귀하가 위 사항에 대하여 동의를 하였더라도 연구가 시작되기 이전에는 언제든지 동의를 철회할 수 있고, 유전자검사의 결과(연구결과는 제외)는 10년간 보존되며, 유전자검사결과, 유전자검사·연구동의서, 유전자연구기관·유전자은행으로의 검사대상물 제공기록은 본인 또는 법정대리인이 요구하는 경우 언제든지 열람할 수 있습니다.  3) 본 기관은 귀하의 개인정보 보호를 위하여 필요한 조치를 취하여야 하며, 모든 연구는 기관생명윤리심의위원회의 심의와 승인을 얻은 후 진행될 것입니다.  4) 귀하의 검사대상물을 이용한 연구결과에 따른 새로운 약품이나 진단도구 등 상품개발 및 특허출원 등에 대해서는 귀하의 권리를 주장할 수 없으며, 검사대상물과 공여한 정보를 이용한 연구는 학회와 학술지에 연구자의 이름으로 발표되고 귀하의 신상은 드러나지 않습니다.  본인은 유전자검사·연구에 대하여 충분한 설명을 들었으며, 위의 사항에 대한 동의는 자발적 의사에 의한 것임을 밝히는 바입니다. | | | | | | |
| 서명 년 월 일 검사·연구대상자 법정대리인  상담자 | | | | | | |
| ※ 구비서류: 법정대리인의 경우 법정대리인임을 증명하는 서류 | | | | | | |
| 210mm×297mm(일반용지 60g/㎡(재활용품)) | | | | | | |
