# Supplementary material for: The Impact of Oxytocin on Food Intake and Emotion Recognition in Patients with Eating Disorders: A Double Blind Single Dose Within-Subject Cross-Over Design
Source: PLoS One. 2015 Sep 24;10(9):e0137514. doi: 10.1371/journal.pone.0137514 (PMC4581668; doi:10.1371/journal.pone.0137514)
Supplement: S3 Text — (DOC) [file pone.0137514.s004.doc]

Oxytocin system as the mechanism of social emotional

dysfunction in eating disorder

Seoul Paik Hospital Inje University

Department of Neuropsychiatry,

Youl-Ri Kim

Version 1.3

September 27, 2012

**Contents**

Synopsis

1. Project title

2. Background

3. Objectives

4. Place, Duration, and Researchers

5. Participants

6. Sample size

7. Informed consent

8. Method

A. Questionnaire-Based Measures of Psychopathology

B. Measurements

C. Intervention

D. Analysis of methylation status of the OXTR promoter region

9. Safety of oxytocin : Provided in method

10. Process of the study : Provided in method

11. Statistical methods : Provided in method

12. Compensation for any damage : Provided in method

13. Data protection : Provided in method

14. References

**Synopsis**

| **Purpose** | The aim of this study is to investigate oxytocin system as the mechanism of social emotional dysfunction in eating disorders. |
| --- | --- |
| **Participant** | 50 women with eating disorders (ages 12~65 years),  50 healthy women (ages 12~65 years) |
| **Contents** | This project consists of 2 studies; 1) to obtain genetic evidence for the role of oxytocin receptor gene in eating disorders, 2) to examine the impact of oxytocin on the social emotional functioning in eating disorders. |
| **Method** | This project is conducted with double blind placebo controlled cross-over design.  This project is conducted through two times. In first visit, the participant is injected with either oxytocin(40IU, between 18-24IU for ages 12~15 years) or placebo. After nasal application, the participant conducts interview and questionnaire. In second visit at least 1 week after, the participant is injected with the other one(oxytocin or placebo) and conduct same procedure with first visit. |
| **Statistical Analysis** | The main dependent variables were accuracy and emotional intensity. Corrected emotional intensity was calculated from the relative emotional intensity at the point of identification of an emotion. Neutral faces (0% relative emotional intensity) were developed into full-blown expressions (100% relative emotional intensity) in 50 stages, with an increase in intensity of 2% in each frame. The corrected emotional intensity conceptualizes the emotional intensity as observed by the participant, with 100 as the strongest possible intensity of a given emotion. A low emotional intensity required to identify an emotion indicates sensitivity to that emotion, and high intensity indicates insensitivity. Accuracy refers the correct categorization of the emotion. |

**1. Project title**

Oxytocin system as the mechanism of social emotional dysfunction in eating disorder

**2. Background**

**Introduction**

Eating disorders affect approximately 2% of the population, of which anorexia nervosa accounts for 1% (Hudson *et al.*, 2007) of all cases. The average duration of anorexia nervosa is 7 years; 25% of those affected remain ill for life (Vos *et al.*, 2001), dependent on benefits, families and repeated hospital care(Hjern *et al.*, 2006), and have premature deaths (Harris and Barraclough, 1998). The main reasons for the relative lack of efficacy of the current treatment methods of anorexia nervosa is that most of them have been adapted from the procedures developed for other disorders and are neither tailored to the unique characteristics and needs of people with anorexia nervosa, nor focused on how the disorder is maintained.

**Emotional/social phenotype in eating disorder**

People with eating disorders have a wide variety of problems with social and emotional functioning, which have been detailed in systematic reviews (Oldershaw *et al.*, 2011; Zucker *et al.*, 2007). In a longitudinal cohort study, a subgroup with marked problems with empathy with autistic spectrum disorder (ASD) traits had a poor prognosis (Gillberg *et al.*, 2010 ; Wentz *et al.*, 2009). We found that people with eating disorders have an attentional bias to negative emotional faces and away from positive faces (Cardi V and Treasure J, 2011; Cserjesi R *et al.*, 2011 in press). This form of attentional bias is thought to play a causal role in the development and maintenance of anxiety (Harrison *et al.*, 2010) which a common comorbidity in eating disorders and ASD disorders.

**Brain activation related to food and eating**

Reduced activation of the basal ganglia and cerebellum to food cues has been demonstrated in both currently ill and recovered anorexia nervosa patients compared to healthy people, suggesting that food is less rewarding in the former (Uher *et al.*, 2004; Wagner *et al.*, 2008). There is, however, hyper activation within a network of frontal brain regions including the dorsolateral prefrontal cortex (DLPFC), anterior cingulate cortex, presupplementary motor cortex and anterior insular cortex (Uher *et al.*, 2004), which comprise the circuits associated with the executive function and cognitive control. Activation in this system, particularly the right DLPFC, is heightened in the restricting form of anorexia nervosa, as might be expected from the extreme form of dietary restraint in this form of eating disorder(Brooks, 2010). Those patients who have recovered from anorexia nervosa have a similar pattern of brain activation to those who are currently unwell, but with additional activation in the prefrontal areas and anterior cingulate regions. These may represent new, non-fear learning or extinction circuits that have been developed as part of the recovery process.

**Oxytocin on social emotional functioning**

The interest in oxytocin as a recently proposed hypothesis suggests that the link between autistic spectrum disorders and anorexia nervosa may be explained by a common abnormality in oxytocin function (Odent, 2010). Oxytocin also appears to be a potent modulator in the processing of social stimuli. For example, oxytocin was found to suppress anxiety to psychosocial stress (Heinrichs *et al.*, 2003) and induce a substantial increase in trust (Kosfeld *et al.*, 2005). The role of the oxytocin system in the etiology of autism has been suggested, and the epigenetic regulation of the oxytocin receptor gene has been implicated in the development of autism (Gregory *et al.*, 2009). These findings justified a pilot study of the possible therapeutic effects of oxytocin in autism (Bartz and Hollander, 2008).

There have not been any in-depth inquiries into the oxytocin system of anorexic patients. However, it has been reported that the level of oxytocin in the cerebrospinal fluid of women with ‘restricting anorexia’ is significantly lower than that in bulimic and control subjects(Demitrack *et al.*, 1990). Such studies of the oxytocin system provide new reasons to dismantle the framework of ‘eating disorders’ while reinforcing the links between anorexia and autism. They offer interpretations of the perinatal period as being critical in the origin of both conditions. The administration of oxyctocin may alleviate some of the abnormalities in social functioning in anorexia nervosa patients.

**Oxytocin receptor gene for epigenetic change**

Until recently, research into the genetic etiology of anorexia nervosa yielded few conclusive findings, but a recent study suggests the involvement of not only genetic but also epigenetic factors (Campbell *et al.*, 2011). There has been a variety of genomic and epigenetic evidence for oxytocin receptor gene (*OXTR)* deficiency in autism, whereas genetic investigation of oxytocin system in eating disorders was very few. Gregory et al.(Gregory *et al.*, 2009) identified a deletion in the *OXTR* gene in an autism proband; moreover, the hypermethylation of residues critical for *OXTR* gene silencing was observed in affected siblings, suggesting the epigenetic misreguation of this gene through aberrant gene silencing by DNA methylation. Increases in DNA methylation in the CpG island is known to regulate *OXTR* expression were found not only in the temporal cortex but also in peripheral blood cells, in independent datasets of individuals with autism, compared with control subjects. These data implicate the epigenetic regulation of *OXTR* in the development of autism.

Among the potential epigenetic candidates for anorxia nervosa, *OXTR* silencing by hypermethylation is of particular interest, because of the possible link between AN and autism. Autistic traits such as weak central coherence have also been suggested as an endophenotype of anorexia nervosa (Kim *et al.*, 2011; Lopez *et al.*, 2008), which together with other observations suggests the existence of a genetic link between anorexia nervosa and autism.

The human *OXTR* is a 389-amino-acid polypeptide with seven transmembrane domains and belongs to the class I G protein-coupled receptor family (Gimpl and Fahrenholz, 2001). *OXTR* is located on chromosome 3p25, spans ~19 kbp and contains three introns and four exons (Inoue *et al.*, 1994). The oxytocin receptor, *OXTR*, is spatially and temporally regulated by a complex combination of sex hormones, inflammatory cytokines, oxytocin feedback and epigenetic mechanisms (Kusui *et al.*, 2001). Recent findings (Gregory *et al.*, 2009) suggested that methylation status of the *OXTR* promoter region in the peripheral blood mononuclear cells could reflect an early developmental event prior to germ layer specification, thus providing a surrogate for the methylation status in the temporal cortex. Based on the findings, we hypothesize that the epigenetic regulation of *OXTR* expression may be significantly different between anorexia nervosa and healthy women, that this difference could be detected by analysing the methylation status in peripheral blood cells and that the methylation levels of *OXTR* are correlated with social emotional functioning in anorexia nervosa.

**The psychophysiology related to food and eating**

A recent systematic review summarised the subjective and objective responses to pictures of food in people with eating disorders (Giel *et al.*, 2011). Food images elicit less smiles in people with eating disorder(Soussignan *et al.*, 2010), create an experience of disgust and fear (Jiang *et al.*, 2010; Uher *et al.*, 2004) and do not attenuate the startle response (a form of defensive reaction) (Friederich *et al.*, 2006). Priming with a subliminal presentation of fearful faces exaggerates the negative reaction to food (Soussignan *et al.*, 2010) and systematic reviews of attentional reaction studies suggest a heightened vigilance to food. These findings are consistent with the hypothesis of a conditioned aversion to food in eating disorders.

**Developing new models to explain maintenance of eating disorders**

Considering the psychophysiology related to food and eating in eating disorders and accumulated similarities in anorexia nervosa and autism, we develop a new maintenance model of eating disorders with problematic eating behaviour and impaired socio-emotional functioning as the core components. We hypothesize a conditioned aversion to food in eating disorders based on a psychophysiology related to food and eating. Oxytocin, a potent modulator in the processing of social stimuli, provides new reasons to dismantle the framework of ‘eating disorders’ while reinforcing the links netween anorexia and autism. Oxytocin dysfunction may come from epigenetic misregulation, which develops and maintains eating disorders. We hypothesize that oxytocin may alleviate some of the abnormalities in social functioning in eating disorders. The functional relevance of the neural circuits in the maintenance of eating disorders is inferred from the activation patterns recorded in situations with a high probability of symptom manifestation.

**3. Objectives**

The aim of this study is to develop a new maintenance model of eating disorders with conditioned aversion to food and impaired social emotional functioning as core components with oxytocin dysfunction as a main underlying mechanism. In this project we will conduct a series of studies (1) to obtain genetic and epigenetic evidence for the role of *OXTR* gene in people with eating disorders, (2) to examine the impact of oxytocin on the social emotional functioning.

**4. Place, Duration, and Researchers**

Place: Eating disorders clinic, Seoul Paik Hospital

Duration: 2012. 4. 12 ~ 2013. 4. 11.

Researchers : Principal Investigator Youl-Ri Kim, Researcher Eun-Young Jang, Seung-Min Oh.

**5. Participants**

Patients

The patients with eating disorders will be recruited from both the outpatient clinic and the inpatient ward at the Eating Disorders Clinic of Seoul Paik Hospital, Seoul, South Korea. The inclusion criteria are patients (a) age 12 or over, and (b) having DSM anorexia nervosa or bulimia nervosa. The exclusion criteria include patients with insufficient knowledge to understand the treatment, learning disabilities, severe mental or physical illness needing treatment in its own right (e.g. schizophrenia, schizoaffective, psychosis not otherwise specified or diabetes mellitus), substance dependence, autism spectrum disorder or pregnancy. Patients on antidepressants will be included, provided they are on a stable dose, i.e. for at least 4 weeks.

Healthy controls

Participants in the healthy controls will be recruited from outside institution. The inclusion criteria are (a) a BMI of between 19 and 25 (kg/m2), and (b) no personal or family history of psychiatric illness or eating disorders.

Healthy participants and patients will be tested during the follicular phase of their menstrual cycle (approximately days 3–12).

**6. Sample size**

Based on the results obtained by Guastella et al. (Guastella et al., 2010), N=20 is the chosen sample size for a double-blind, placebo controlled within-subject design, based on mean (SD) of difference = 1.08(0.05) between oxytocin and placebo at the 5% significance level with 80% power. Considering 25% of dropout rate during interval, N=25 will be justified as a sample size in each group. We will recruit 25 patients with anorexia nervosa, 25 patients with bulimia nervosa, as well as the same number of healthy women (N=50) to compare with patients at baseline.

**7. Informed consent**

Attached separate documents

**8.** **Method**

**A. Questionnaire-Based Measures of Psychopathology**

Depression, Anxiety and Stress Scale (DASS): A 21-item self-report measure to assess mood state over the past 7 days using a 4-point Likert scale (Lovibond SH and PF., 1995; Lovibond and Lovibond, 1993 ). Three subscales are used to yield the scores for depression, anxiety and stress. Good reliability and validity is reported (Brown TA et al., 1997). This is not standardized yet in the country, so we will carry the standardization of the questionnaire in Korean also during this project.

**B. Measurements**

a. height and weight

b. food diary for 24 hours

c. the amount of apple juice after experiments

**C. Intervention**

Intranasal oxytocin

In the first step, oxytocin or a placebo (containing all ingredients except for the peptide) will be administered with a nasal spray. It has been shown that neuropeptides pass the blood–brain barrier reliably after intranasal application (Born et al., 2002). Several studies using this method have reported oxytocin-dependent effects on either behaviour or brain function (Baumgartner et al., 2008; Domes et al., 2007a; Kirsch et al., 2005; Kosfeld et al., 2005). The spray will be administered to the participants four times with a delay of 45s between each administration; each administration will consist of one inhalation of the spray into each nostril. Each inhalation will contain approximately 4 IU. The participants will receive a total of 40-IU(between 18-24IU for ages 12~15 years) oxytocin in the oxytocin condition. The session (oxytocin, placebo) order will be randomized using a double-blind procedure. A neuropsychological battery of tests will begin 45 min after spray administration. The outcome will be examined by means of a battery of tests including attentional bias to food and social emotional functioning.

Procedure

Subsequent to comparison of the baseline status of social emotional functioning of patients with healthy controls, we will carry the experiments with a double-blind, placebo-controlled within-subject design. Oxytocin and placebo will be administered intranasally (Born et al., 2002; Domes et al., 2007b; Heinrichs et al., 2003; Kosfeld et al., 2005) with a 1-week interval, forty-five minutes before the neuropsychological tasks. At initial contact, all participants will be screened for a significant degree of anorexic symptomatology using the EDE (Fairburn and Cooper, 1993). In the absence of previous testing, participants will be assessed intelligence by the Korean version of the Wechsler Adult Intelligent Scale (Yum et al., 1992). Participants will complete a medical and psychiatric review with the principal investigator to confirm diagnosis using both DSM-IV assessment criteria and case review. Participants will be instructed to abstain from alcohol and caffeine on the day of drug administration and food and drink (except water) 2 hours before drug administration. After a description of the study, written consent will be obtained from participants. Participants will be told they could withdraw at any time. During the waiting period, participants will complete the EDE-Q (Fairburn and Beglin, 1994), a qualified self-report questionnaire assessing eating disorders symptoms, and DASS, a self report measure to assess depression, anxiety and stress state over the past 7 days (Lovibond and Lovibond, 1993 )

Statistical Analysis

The main dependent variables were accuracy and emotional intensity. Corrected emotional intensity was calculated from the relative emotional intensity at the point of identification of an emotion. Neutral faces (0% relative emotional intensity) were developed into full-blown expressions (100% relative emotional intensity) in 50 stages, with an increase in intensity of 2% in each frame. A low emotional intensity required to identify an emotion indicates sensitivity to that emotion, and high intensity indicates insensitivity. Accuracy refers the correct categorization of the emotion. Analyses will be performed using SPSS version 19. The p-value for two-tailed significance will be 0.05.

**D. Analysis of methylation status of the OXTR promoter region**

To investigate the possibility that eating disorders may be affected by the epigenetic silencing of the OXTR gene, the methylation status of the promoter region of the OXTR gene will be analysed by bisulphite sequencing. DNA isolated from blood will be bisulphite-converted using a methylSEQr Bisulphite Conversion Kit (Applied Biosystems, CA, USA). The promoter region of the OXTR gene including the MT2 region will be PCR-amplified with the bisulphite-treated and non-treated DNA as a template. The PCR primers are: 5’-AAGTTTTGGAATTTTTGATTTG-3’ and 5’-CCAATAAAAAACCTCAACTTAAC-3’. PCR will be performed using the following reaction conditions, 1 cycle: 95°C for 3 min, 55 cycles: 95°C for 1 min followed by 56.8°C for 1 min followed by 72°C for 3 min, and a final extension at 72°C for 5 min. The PCR products will be electrophoresed on agarose gel, purified with a Gel Extraction Kit (Qiagen, CA, USA) and cloned into the TOPO TA vector (Invitrogen, CA, USA). Ten individual clones from each sample are to be sequenced to calculate percentage methylation at each of the CpG sites. The bisulphite conversion will be verified for each clone by assessing the C to T conversion of non-CpG sites. The Welch-Satterthwaite t-test will be employed to adjust for unequal variances between groups and to compare the mean methylation level (%) at each site between the patients (anorexia nervosa and bulimia nervosa) and control groups.

Statistical Analysis

The average methylation level (%) will be determined for each individual and these values will be used to generate an average percentage methylation for each group, anorexic cases and normal controls, respectively. The Welch-Satterthwaite t-test will be employed to adjust for unequal variances between groups and to compare the mean methylation level (%) at each site between the anorexic cases versus control groups. A nominal significance of P < 0.05 indicated a significant change in the methylation state between the two groups. The direction of the change will be determined by comparing the average methylation level at each site between the two groups.

**9. Data Protection**

The data used for study is only accessible to researchers involving the study working within the ED Clinic at the Seoul Paik Hospital who have completed --- (certificate). All paper and electronic data resulting from this research will be stored securely. The database is only accessible to the researchers and is password protected. Any information and data related to the research will be keep confidential.

**References**

Barrett, J. C., Fry, B., Maller, J. and Daly, M. J. (2005) Haploview: analysis and visualization of LD and haplotype maps. *Bioinformatics* **21,** 263-265.

Bartz, J. A. and Hollander, E. (2008) Oxytocin and experimental therapeutics in autism spectrum disorders. In: *Advances in Vasopressin and Oxytocin: From Genes to Behaviour to Disease*. pp. 451-462. Eds. I. D. Neumann, R. Landgraf.

Baumgartner, T., Heinrichs, M., Vonlanthen, A., Fischbacher, U. and Fehr, E. (2008) Oxytocin shapes the neural circuitry of trust and trust adaptation in humans. *Neuron* **58,** 639-650.

Born, J., Lange, T., Kern, W., McGregor, G. P., Bickel, U. and Fehm, H. L. (2002) Sniffing neuropeptides: a transnasal approach to the human brain. *Nature Neuroscience* **5,** 514-516.

Brooks (2010) An investigation of the neural processes associated with eating disorder symptomatology. University of London: London.

Brooks, S., Prince, A., Stahl, D., Campbell, I. C. and Treasure, J. (2011) A systematic review and meta-analysis of cognitive bias to food stimuli in people with disordered eating behaviour. *Clinical Psychology Review* **31,** 37-51.

Brown TA, Chorpita BF, Korotitsch W and DH., B. (1997) Psychometric properties of the Depression Anxiety Stress Scales (DASS) in clinical samples. *Behaviour Research and Therapy* **35,** 79-89.

Campbell, I. C., Mill, J., Uher, R. and Schmidt, U. (2011) Eating disorders, gene-environment interactions and epigenetics. *Neuroscience and Biobehavioral Reviews* **35,** 784-793.

Cardi V and Treasure J (2011) Social reward and rejection sensitivity in Eating Disorders: an investigation of attentional bias and early experiences. *Biological Psychiatry*.

Cserjesi R, Vermeulen N, Lenard L and O., L. (2011 in press) Reduced capacity in automatic processing of facial expression in restrictive anorexia nervosa and obesity. *Psychiatry Research*.

Davies H, S. U., Stahl D, Tchanturia K. (2011 in press) Evoked facial emotional expression and emotional experience in people with anorexia nervosa. *International Journal of Eating Disorders*.

Demitrack, M. A., Lesem, M. D., Listwak, S. J., Brandt, H. A., Jimerson, D. C. and Gold, P. W. (1990) CSF oxytocin in anorexia nervosa and bulimia nervosa -clinical and pathophysiologic considerations *American Journal of Psychiatry* **147,** 882-886.

Domes, G., Heinrichs, M., Glascher, J., Buchel, C., Braus, D. F. and Herpertz, S. C. (2007a) Oxytocin attenuates amygdala responses to emotional faces regardless of valence. *Biological Psychiatry* **62,** 1187-1190.

Domes, G., Heinrichs, M., Michel, A., Berger, C. and Herpertz, S. C. (2007b) Oxytocin improves "mind-reading" in humans. *Biological Psychiatry* **61,** 731-733.

Fairburn, C. G. and Beglin, S. J. (1994) Assessment of eating disorders - interview or self-report questionnaire. *International Journal of Eating Disorders* **16,** 363-370.

Fairburn, C. G. and Cooper, Z. (1993) *The Eating Disorders Examination (12th edition). Binge eating: Nature, assessment and treatment.* Guilford: New York, NY.

Friederich, H. C., Kumari, V., Uher, R., Riga, M., Schmidt, U., Campbell, I. C., Herzog, W. and Treasure, J. (2006) Differential motivational responses to food and pleasurable cues in anorexia and bulimia nervosa: a startle reflex paradigm. *Psychological Medicine* **36,** 1327-1335.

Giel, K. E., Teufel, M., Friederich, H.-C., Hautzinger, M., Enck, P. and Zipfel, S. (2011) Processing of pictorial food stimuli in patients with eating disorders—A systematic review. *International Journal of Eating Disorders***,** Epub ahead of print, DOI: 10.1002/eat.20785.

Gillberg, I. C., Billstedt, E., Wentz, E., Anckarsater, H., Rastam, M. and Gillberg, C. Attention, executive functions, and mentalizing in anorexia nervosa eighteen years after onset of eating disorder. *Journal of Clinical and Experimental Neuropsychology* **32,** 358-365.

Gimpl, G. and Fahrenholz, F. (2001) The Oxytocin Receptor System: Structure, function, and regulation. *Physiological Reviews* **81,** 629-683.

Gotlib, I. H., Krasnoperova, E., Yue, D. N. and Joormann, J. (2004) Attentional biases for negative interpersonal stimuli in clinical depression. *Journal of Abnormal Psychology* **113,** 127-135.

Gregory, S. G., Connelly, J. J., Towers, A. J., Johnson, J., Biscocho, D., Markunas, C. A., Lintas, C., Abramson, R. K., Wright, H. H., Ellis, P., Langford, C. F., Worley, G., Delong, G. R., Murphy, S. K., Cuccaro, M. L., Persico, A. and Pericak-Vance, M. A. (2009) Genomic and epigenetic evidence for oxytocin receptor deficiency in autism. *BMC Medicine* **7**.

Guastella, A. J., Einfeld, S. L., Gray, K. M., Rinehart, N. J., Tonge, B. J., Lambert, T. J. and Hickie, I. B. (2010) Intranasal Oxytocin Improves Emotion Recognition for Youth with Autism Spectrum Disorders. *Biological Psychiatry* **67,** 692-694.

Harris, E. C. and Barraclough, B. (1998) Excess mortality of mental disorder. *British Journal of Psychiatry* **173,** 11-53.

Harrison, A., Tchanturia, K. and Treasure, J. (2010) Attentional Bias, Emotion Recognition, and Emotion Regulation in Anorexia: State or Trait? *Biological Psychiatry* **68,** 755-761.

Heinrichs, M., Baumgartner, T., Kirschbaum, C. and Ehlert, U. (2003) Social support and oxytocin interact to suppress cortisol and subjective responses to psychosocial stress. *Biological Psychiatry* **54,** 1389-1398.

Hjern, A., Lindberg, L. and Lindblad, F. (2006) Outcome and prognostic factors for adolescent female in-patients with anorexia nervosa: 9-to 14-year follow-up. *British Journal of Psychiatry* **189,** 428-432.

Hudson, J. I., Hiripi, E., Pope, H. G. and Kessler, R. C. (2007) The prevalence and correlates of eating disorders in the national comorbidity survey replication. *Biological Psychiatry* **61,** 348-358.

Inoue, T., Kimura, T., Azuma, C., Inazawa, J., Takemura, M., Kikuchi, T., Kubota, Y., Ogita, K. and Saji, F. (1994) Structural organization of the human oxytocin receptor gene. *Journal of Biological Chemistry* **269,** 32451-32456.

Jiang, T., Soussignan, R., Rigaud, D. and Schaal, B. (2010) Pleasure for visual and olfactory stimuli evoking energy-dense foods is decreased in anorexia nervosa. *Psychiatry Research* **180,** 42-47.

Kim, Y. R., Lim, S. J. and Treasure, J. (2011) Different Patterns of Emotional Eating and Visuospatial Deficits Whereas Shared Risk Factors Related with Social Support between Anorexia Nervosa and Bulimia Nervosa. *Psychiatry Investigation* **8,** 9-14.

Kirsch, P., Esslinger, C., Chen, Q., Mier, D., Lis, S., Siddhanti, S., Gruppe, H., Mattay, V. S., Gallhofer, B. and Meyer-Lindenberg, A. (2005) Oxytocin modulates neural circuitry for social cognition and fear in humans. *Journal of Neuroscience* **25,** 11489-11493.

Kosfeld, M., Heinrichs, M., Zak, P. J., Fischbacher, U. and Fehr, E. (2005) Oxytocin increases trust in humans. *Nature* **435,** 673-676.

Kusui, C., Kimura, T., Ogita, K., Nakamura, H., Matsumura, Y., Koyama, M., Azuma, C. and Murata, Y. (2001) DNA methylation of the human oxytocin receptor gene promoter regulates tissue-specific gene suppression. *Biochemical and Biophysical Research Communications* **289,** 681-686.

Lerer, E., Levi, S., Salomon, S., Darvasi, A., Yirmiya, N. and Ebstein, R. P. (2008) Association between the oxytocin receptor (OXTR) gene and autism: relationship to Vineland Adaptive Behavior Scales and cognition. *Molecular Psychiatry* **13,** 980-988.

Lopez, C., Tchanturia, K., Stahl, D. and Treasure, J. (2008) Central coherence in eating disorders: a systematic review. *Psychological Medicine* **38,** 1393-1404.

Lovibond, S. H. and Lovibond, P. F. (1993 ) *Manual for the Depression Anxiety Stress Scales (DASS)*. Psychology Foundation: Sydney.

Macleod, C., Mathews, A. and Tata, P. (1986) Attentional bias in emotional disorders. *Journal of Abnormal Psychology* **95,** 15-20.

Mansell, W., Clark, D. M., Ehlers, A. and Chen, Y. P. (1999) Social anxiety and attention away from emotional faces. *Cognition & Emotion* **13,** 673-690.

Mogg, K. and Bradley, B. P. (1998) A cognitive-motivational analysis of anxiety. *Behaviour Research and Therapy* **36,** 809-848.

Odent, M. (2010) Autism and anorexia nervosa: Two facets of the same disease? *Medical Hypotheses* **75,** 79-81.

Oldershaw, A., Hambrook, D., Stahl, D., Tchanturia, K., Treasure, J. and Schmidt, U. (2011) The socio-emotional processing stream in Anorexia Nervosa. *Neuroscience and Biobehavioral Reviews* **35,** 970-988.

Rabinowitz, D. and Laird, N. (2000) A unified approach to adjusting association tests for population admixture with arbitrary pedigree structure and arbitrary missing marker information. *Human Heredity* **50,** 211-223.

Sham, P. C. and Curtis, D. (1995) An extended transmission/disequilibrium test (TDT) for multiallele marker loci. *Annals of Human Genetics* **59,** 323-336.

Soussignan, R., Jiang, T., Rigaud, D., Royet, J. P. and Schaal, B. (2010) Subliminal fear priming potentiates negative facial reactions to food pictures in women with anorexia nervosa. *Psychological Medicine* **40,** 503-514.

Uher, R., Murphy, T., Brammer, M. J., Dalgleish, T., Phillips, M. L., Ng, V. W., Andrew, C. M., Williams, S. C. R., Campbell, I. C. and Treasure, J. (2004) Medial prefrontal cortex activity associated with symptom provocation in eating disorders. *American Journal of Psychiatry* **161,** 1238-1246.

Vos, T., Mathers, C., Herrman, H., Harvey, C., Gureje, O., Bui, D., Watson, N. and Begg, S. (2001) The burden of mental disorders in Victoria, 1996. *Social Psychiatry and Psychiatric Epidemiology* **36,** 53-62.

Wagner, A., Aizenstein, H., Mazurkewicz, L., Fudge, J., Frank, G. K., Putnam, K., Bailer, U. F., Fischer, L. and Kaye, W. H. (2008) Altered insula response to taste stimuli in individuals recovered from restricting-type anorexia nervosa. *Neuropsychopharmacology* **33,** 513-523.

Watson, D., Clark, L. A. and Tellegen, A. (1988) Development and validation of brief measures of positive and negative affect - the PANAS scales *Journal of Personality and Social Psychology* **54,** 1063-1070.

Wentz, E., Gillberg, I. C., Anckarsater, H., Gillberg, C. and Rastam, M. (2009) Adolescent-onset anorexia nervosa - missing half of the story? Reply. *British Journal of Psychiatry* **194,** 565-565.

Wu, S. P., Jia, M. X., Ruan, Y., Liu, J., Guo, Y. Q., Shuang, M., Gong, X. H., Zhang, Y. B., Yang, X. L. and Zhang, D. (2005) Positive association of the oxytocin receptor gene (OXTR) with autism in the Chinese Han population. *Biological Psychiatry* **58,** 74-77.

Yum, T. H., Park, Y. S., Oh, K. J., Kim, J. G. and Lee, H. Y. (1992) *The manual of Korean-Wechsler adult intelligence scale*. Korean Guidance Press: Seoul.

Zucker, N. L., Losh, M., Bulik, C. M., Labar, K. S., Piven, J. and Pelphrey, K. A. (2007) Anorexia nervosa and autism spectrum disorders: Guided investigation of social cognitive endophenotypes. *Psychological Bulletin* **133,** 976-1006.
