# Supplementary material for: The Impact of Oxytocin on Food Intake and Emotion Recognition in Patients with Eating Disorders: A Double Blind Single Dose Within-Subject Cross-Over Design
Source: PLoS One. 2015 Sep 24;10(9):e0137514. doi: 10.1371/journal.pone.0137514 (PMC4581668; doi:10.1371/journal.pone.0137514)
Supplement: S4 Text — (DOC) [file pone.0137514.s005.doc]

섭식장애 유지모델로서 사회 정서기능의 이상 및

옥시토신 메카니즘의 이상 규명

인제대학교 서울백병원

정신건강의학과

김 율 리

Version 1.3

2012년 9월 27일

**목 차**

연구계획서 요약

1. 연구 제목

2. 연구의 배경

3. 연구 목적

4. 연구 수행 장소, 기간 및 연구자

5. 대상자 선정기준

A. 선정기준 (inclusion criteria)

B. 제외기준 (exclusion criteria)

C. 중지 탈락 기준

6. 대상 피험자의 수

A. 목표 피험자의 수

B. 산출근거

7. 대상 환자의 시험참가 동의서

A. 첨부 1

8. 평가하여야 할 항목

A. 자가보고식 평가

B. 측정 항목

C. 옥세토신 조제 및 투여

D. 신경심리학적 평가

9. 옥시토신의 특성 및 비강내 투여의 안정성

A. 옥시토신 특성 및 안전성

B. 옥시토신 비강 내 투여의 안전성 (Macdonald 등 2011)

10. 연구실행계획표

A. 연구설계

B. 실험절차

11. 통계처리방법

12. 피해에 대한 보상

13. 자료관리 및 개인정보보안

14. 참고문헌

**연구계획서 요약**

| **연구목표** | 본 연구는 섭식장애에서의 이상섭식행동 및 손상된 사회/정서기능의 메커니즘을 규명하고, 섭식장애에서의 옥시토신 기능장애 가설을 검증하고자 함. |
| --- | --- |
| **대상**  **피험자 및 수** | 섭식장애 환자 50명 및 정상대조군 여성50명 |
| **연구내용** | 본 연구과제에서는 통제된 실험을 통해 섭식장애 환자의 음식에 대한 불안 및 사회/정서적 결함에 대한 옥시토신의 영향을 규명하고자 함. 구체적으로는 인체호르몬인 옥시토신 투여 후 신경인지검사를 통해 섭식장애 환자들의 음식에 대한 주의(attention)의 편향(bias)및 사회/정서적 인식 기능의 변화를 알아볼 것임. |
| **연구방법** | 본 연구는 무작위배정, 이중 맹검, 크로스오버(cross-over)방식으로 진행함. 본 연구는 약 1주일 간격을 두고 2일에 걸쳐 시행함. 첫 방문에서, 피험자는 옥시토신 (40 IU, 단 12-15세의 경우 18-24IU 범위에서 적합한 용량 선택) 과 위약 중 하나를 투여 받은 후 섭식 및 정서상태의 변화를 평가하는 신경심리검사를 시행함. 두 번째 방문에서는 첫날과 다른 시약(옥시토신 혹은 위약)을 투여 받은 후 동일한 절차를 수행함. |
| **통계분석** | 1) 신경인지검사(Dot-probe task)에 대한 자료 분석  옳은 반응(correct responses)에 대한 반응시간(reaction time; RT)에 기초할 것임. RT 가 200 ms 미만이거나 2,000 ms 이상인 경우는 제외할 것임. 편향점수 (bias score)는 다음과 같이 계산함. [편향점수 = RT: 표적그림(target)과 단서(probe) 가 반대 방향; 편향점수 = (-) RT: 표적그림과 단서가 같은 방향; +: 단서가 섭식장애 관련 표적그림과 같은 방향일 때 단서에 더 빨리 반응한 경우; -: 단서가 섭식장애 관련 표적그림과 같은 방향일 때 단서에 더 느리게 반응한 경우]. 3가지 다른 자극 (즉, 긍정, 부정, 중립) 및 2가지 단서의 위치(표적그림과 같은 방향 혹은 반대 방향)에 대해 repeated measures ANOVAs 를 시행 함(이후 paired t-tests).  2) 옥시토신과 위약 간 비교 분석  Repeated measures ANOVAs 를 시행할 것임. 본 연구에서는 그룹(옥시토신, 위약)과 표적그림(target) 간 상호작용이 있을 것이라는 가설 하에 옥시토신이 긍정자극에 대한 반응을 향상시킬 것으로 예상함. |

**1. 연구제목**

섭식장애 유지모델로서 사회 정서기능의 이상 및 옥시토신 메카니즘의 이상 규명

**2. 연구의 배경**

**섭식장애의 치료 현황**

섭식장애를 가진 인구는 전체 인구 중 약 2%를 차지하며, 그 중 신경성 식욕부진증은 1%(Hudson et al., 2007)를 차지한다. 신경성 식욕부진증의 평균 이환 기간은 7년이다. 신경성 식욕부진증 인구 중 25%는 평생 동안 병을 지니고 살아가며(Vos et al., 2001), 가족, 반복적인 병원 치료, 의료보호에 의존하며(Hjern et al., 2006), 일찍 생을 마감한다(Harris and Barraclough, 1998). 최근 신경성 식욕부진증에서 낮은 치료 성과의 주요 원인들로 다른 질병 치료를 위해 개발한 치료 절차의 사용 및, 신경성 식욕부진증 환자의 독특한 특성과 요구의 고려부족, 질병이 어떻게 지속되는지에 대해서 고려 부족 등 때문으로 생각되고 있다.

**옥시토신과 사회 정서 기능**

최근 옥시토신에 대한 관심은 자폐증 및 신경성 식욕부진증에서 공통된 비정상적인 옥시토신 기능을 잘 설명할 수 있을 것이라고 생각되어 각광받고 있다(Odent, 2010). 또한 옥시토신은 사회적 자극 처리 절차를 매우 잘 예측할 수 있는 조절인자로 판명되었다. 예를 들어, 옥시토신이 심리적 스트레스에 대해서 불안을 억제하고(Heinrichs et al., 2003), 신뢰감을 증가시킬 수 있다는 것이 발견되었다(Kosfeld et al., 2005). 자폐증 및 신경성 식욕부진증의 병인론으로써 옥시토신의 역할이 제안되고 있으며, 이상의 발견들을 토대로 자폐증에서 옥시토신의 치료적 효과의 가능성에 대해서 예비연구가 시행된 바 있다 (Bartz & Hollander, 2008).

**음식 및 섭식행동과 관련된 정신 생리**

최근 체계적 문헌 리뷰를 통해 섭식장애가 있는 사람의 음식 그림에 대한 주관적이고, 객관적인 반응을 정리하였다(Giel et al., 2011). 이에 따르면 섭식장애가 있는 사람들은 음식 그림을 보고 덜 웃는다(Soussignan et al., 2010). 음식 그림은 그들에게 혐오감과 두려움을 유발하며(Jiang et al., 2010; Uher et al., 2004), 놀람 반응(방어의 형태)을 초래한다(Friederich et al., 2006). 신경성 식욕부진증에서 주의력의 상승 반응에 관한 연구를 체계적으로 검토해 본 결과 음식에 대한 경계가 고양됨을 암시한다. 이러한 결과들은 섭식장애 환자들에서 음식에 대한 조건화된 혐오감 가설과 일치한다.

**섭식장애에서 정서적/사회적 표현형**

섭식장애가 있는 사람들은 사회적, 정서적 기능에 매우 다양한 문제가 있으며, 이에 대한 내용은 최근의 일련의 체계적 리뷰(systematic reviews)에서 자세히 기술된 바 있다(Oldershaw et al., 2011; Zucker et al., 2007). 장기적인 코호트 연구에서, 공감에 뚜렷한 문제가 있는 자폐증적 (아스퍼거 증후군) 특성을 지니는 하위 그룹은 좋지 못한 예후를 보였다(Gillberg et al., 2010; Wentz et al., 2009). 우리는 섭식장애가 있는 사람들이 부정적인 얼굴 표정에 대해서 주의 편향을 하고, 긍정적인 얼굴 표정에 대해서는 주의를 하지 않는다는 것을 발견했다(Cardi V & Treasure J, 2011; Cserjesi R et al., 2011 in press). 이러한 형태의 주의 편향은 불안의 증가와 유지에 중요한 원인이 되며(Harrison et al., 2010), 불안은 섭식장애와 자폐증의 공통적인 공병요인이다.

**옥시토신 투여의 사회성 호전 효과 및 치료적 가능성** (Striepens, Kendrick, Maier & Hurlemann, 2011).

Striepens 등의 (2011) 체계적 문헌검토 결과 선행연구들에서 체내 옥시토신농도와 사회성 및 친밀 행동 간의 관련성이 입증되었다. 체내 옥시토신 투여 후 친밀한 행동, 보상행동, 사회적 정보에 대한 인식이 강화된다는 증거들이 쌓이고 있다. 한편, 옥시토신 투여는 12-19세의 청소년에서도 안전함이 입증되었으며, 12-15세의 경우에는 18IU 를 사용하였다 (Guastella et al., 2010).

**3. 연구 목적**

본 연구의 목표는 옥시토신의 기능 장애를 기반으로 한 메커니즘과 함께 음식에 대한 조건화된 혐오감과 손상된 사회 정서 기능을 핵심 요소로 하여 섭식장애를 지속시키는 새로운 모델을 만드는 것이다. 이 연구과제를 통해 섭식장애 환자에서 사회 정서 기능에 대한 옥시토신의 영향을 규명한다.

**4. 연구 수행 장소, 기간 및 연구자**

장소: 서울시 중구 저동 2가 85번지 서울백병원 정신건강의학과

기간: 식약청 승인일 부터 2013.12.31 까지

연구담당자: 연구책임자 - 김율리

연구담당자 - 이수진, 한동화, 배도희

관리약사 - 정경희, 강균화

**5. 대상자 선정기준**

**A. 선정기준 (inclusion criteria)**

본 연구는 서울백병원 섭식장애클리닉에 내원한 섭식장애 환자들과 정상 성인들을 대상으로 할 것이다. 대상자 선정기준은 섭식장애의 호발 연령대가 12-15세임을 고려하여 12세 - 65세 사이의 DSM의 섭식장애 아형 중 어느 한 가지 진단기준을 만족하는 경우이다. 옥시토신 투여 효과의 과학적 평가를 위한 대조군은 서울시내 소재 여자대학 심리학과 내부 광고를 통해 건강한 대학생 및 대학원생을 모집할 것이다. 정상군의 경우 자기보고식 설문지를 통해 주요우울증, 양극성장애, 공황장애, 정신증 관련 장애, 물질의존, 간질, 섭식장애, 자페 스펙트럼 장애, 외상성 뇌손상 등의 과거력을 확인하고 해당되는 경우 연구대상에서 제외된다. 또한 모든 연구참가자들이 생리주기상 여포기(생리시작일로부터 약 3-12일 사이)에 본 연구에 참여할 수 있도록 할 것이다. 연구에 참여한 환자 및 정상 성인들에게는 소정의 교통비가 지급될 예정이다.

**B. 제외기준 (exclusion criteria)**

- 연령이 12세 미만이거나 65세 이상

- 청력 및 시각의 유의한 이상

조현병 (정신분열병. 분열형정동장애, 달리 분류되지 않는 정신증), 1형 조울병, 물질사용장애, 자폐스펙트럼 장애 등의 정신증적 질환을 동반한 경우

- 지난 2주 동안 정신상태에 영향을 미칠 수 있는 약물이나 경구피임약을 복용한 경우 (fluoxetine 의 경우 지난 5주), 혹은 내과적 치료 약물을 복용하고 있는 경우

- 현재 임신 혹은 수유 중인 여성

- 연구자가 정신적 인지적 상태가 미약하여 연구를 수행할 수 있지 않다고 판단한 자

**C. 중지 탈락 기준**

- 피험자의 생체증후에 급격한 이상을 보일 경우

- 그 외 연구자가 판단하여 연구를 지속하는 것이 적절치 않다고 판단될 경우

**6. 대상 피험자의 수**

**A. 목표 피험자의 수**

- 총 모집 대상자는 환자 50명 및 정상 성인 50 명임.

**B. 산출근거**

- 귀무가설 (H0) : oxytocin 과 placebo 처치 별로 측정한 정서에 차이가 없음.

- 대립가설 (H1) : oxytocin 과 placebo 처치 별로 측정한 정서에 차이가 있음.

- 독립변수 : 처치 (oxytocin 과 placebo)

- 종속변수 : 정서

- 유의수준 : 예상되는 차이 d = 0.05

- 양측검정, 검정력: Zα = 1.96, α = 0.05, 1-β = 0.8, Zβ = 0.84

- 목표한 피험자의 수 및 근거

Naber et al., (2010) 등이 정상인을 대상으로 한 연구에 의하면, oxytocin 처치군에서 긍정적 정서에 대한 인식능력이 3.96(0.46) 로써, placebo 처치군 3.60(0.59) 에 비해 유의하게 높았다 [F(1,16) = 7.73, p = .01, η^2^ = .33]. 따라서 두 군간 차이를 도출하기 위한 표본수는 본 연구에서 sample size per group for comparing two means 에 의하여 two-tailed α = 0.05, β = 0.20 로 가정하면 최소35명의 환자가 필요하다. 탈락율을 고려하여 본 연구에서는 환자 50 명을 등록하며 동수의 대조군을 함께 모집한다.

**7. 대상 환자의 시험참가 동의서**

**A. 첨부 1**

**연구 1. 섭식장애의 정서 및 섭식병리 이상과 옥시토신**

**8.** **연구방법**

**A. 자가보고식 평가 (self-report questionnaires)**

a. Eating Disorders Examination Questionnaire (EDE-Q) (Fairburn, 1994)

c. 자폐증지수검사 (Autism Spectrum Quotinent; Baron-Cohen et al., 2001)

d. 행동억제/행동활성 척도 (Behavioral inhibition scale/ Behavioral activation scale; BAS/BIS)

e. 긍정부정정서척도 (Positive and Negative Affect Scale; PANAS, Watson et al., 1988)

f. 정서조절곤란척도 (DERS Difficulties in Emotional regulation Scale) (Gratz와 Roemer, 2004)

g. 우울불안스트레스척도 (DASS Depression Anxiety Stress Scales) (Brown et al., 1997)

**B. 측정 항목**

a. 키 및 체중

b. 1일간의 식사일기

c. 실험 후 섭취한 음료의 양

**C. 옥시토신 조제 및 투여**

옥시토신 조제방법은 Marsh 등 (2009)의 방법에 따라 조제할 것이다. 이는 옥시토신 주사제 생산을 허가받은 중외제약㈜에서 위탁제조를 통해 바이알 형식으로 공급받을 것이다. 옥시토신제재는 35.2mg 의 옥시토신(568IU) 원액을 0.9% sodium chloride 용액 300mL 과 혼합한 후 10배 희석한 acetic acid 의 pH 에 맞춘다(pH = 4.01). 이 용액을 멸균한 후 각각의 바이알에 나누어 담는다 (각 2.5ml). 바이알을 냉동시켜 보관 한 후 연구 당일 해동시켜 냉장한다 (4°C). 관리약사는 연구당일 옥시토신 혹은 플라시보를 바이알로부터 비강스프레이로 옮겨 담는다. 연구자는 각 흡입기를 연구참가자에게 제공하고 흡입기 사용법을 교육하여 연구참가자가 자가로 흡입하는 것을 면밀히 모니터한다.

**D. 신경심리학적 평가**

a. 음식 및 불안에 대한 주의 편향 실험 (수정된 dot-probe 과제)

이 검사는 자극 (target)에 대한 과도한 경계 및 회피로 인한 주의편향 (attention bias) 을 측정하기 위함이다. 자극에 대한 느린 반응 시간은 자극에 대한 회피를 나타낸다고 볼 수 있으며, 반면, 빠른 반응 시간은 자극에 대한 과 경계를 나타낸다고 볼 수 있다. 본 연구에서는 섭식, 체형, 체중과 관련된 그림이 타겟 감정 자극이 되게끔 섭식장애에 맞는 자극을 사용하여 실험을 진행 할 것이다. 자극은 섭식관련 사진, 체형관련 사진, 체중관련 사진들을 감정 반응과 어울리는 통제 그림 (비표적 그림들 -동물)과 짝을 지워 실험을 진행할 것이다.

실험 절차는 다음과 같다. 참가자들은 컴퓨터 화면 중앙에 시선이 가도록 앉아서 표적을 응시한다. 모든 그림은 JPEG 컴퓨터 그림 파일로 만들 것이다. 그림을 보여주기 위해 컴퓨터 본체와 51cm 모니터를 연결한다. 모니터 화면의 배경 색은 흰색으로 한다. 표적 그림 쌍(섭식, 체형, 체중)과 비표적 그림들(동물)이 화면의 왼쪽과 오른쪽에 나타난 후 두 그림이 화면에서 사라지면, 연이어 점이 나타날 것이다. 점이 사라진 직후 참가자들은 점의 위치가 오른쪽인지 왼쪽인지를 나타내기 위해 키보드에서 맞는 버튼을 누르게 된다. 반응을 하고 난 후에 점은 사라지고, 바로 다음 그림이 나타날 것이다. 참가자는 1인씩 단독으로 검사를 받고, 옥시토신과 위약의 처치 상황을 모르는 연구자는 참가자에게 가능한 빠르고 정확하게 검사를 하게끔 지시한다.

b. 얼굴표정인식 - Facial expression morphing 과제

얼굴표정인식과제로 양재원(2009)이 개발한 정서인식과제이다. 컴퓨터로 실시하며, 모니터에 제시되는 얼굴표정을 보고 변별하는 방식으로 진행된다. 실험은 상향(ascending)과 하향(descending) 실험의 두 부분으로 나누어져 있다. 상향실험에서는 모니터 중앙에 응시점이 500ms동안 제시된 후 중립의 얼굴표정이 제시되며 이 때 얼굴 표정이 중립에서 시작하여 점차로 특정 정서를 가진 표정으로 변화하게 된다. 피험자는 제시되는 얼굴표정사진을 바라보다가 변화하는 얼굴표정에서 어떤 정서를 감지하게 되면 스페이스 바를 눌러 반응해야 한다. 피험자가 반응을 하면 얼굴표정은 더 이상 변화하지 않고 모니터에서 사라지며, ‘제시된 얼굴표정의 정서가 무엇인지’를 묻는 질문이 나타나도록 하였다. 이 때 피험자는 0∼3의 숫자 키보드를 이용하여, ‘슬픈’, ‘두려운’, ‘화난’, ‘기쁜’ 중 하나의 반응을 선택하게 된다. 본 시행 24회로 구성되어 있으며, 본 과제의 총 소요시간은 20분 정도이다.

c. 신뢰게임 (trust game)

신뢰 게임이란 사회 경제적 상호 작용 상황에서 상대에 대한 신뢰가 의사 결정에 미치는 영향을 알아보기 위해 실험 상황에서 많이 활용되는 방법 중의 하나이다. 두 명의 참가자가 각각 투자자(investor)와 수탁자(trustee) 역할을 수행하여, 상호신뢰를 토대로 투자금액을 증가시키는 것을 평가하는 방법이다 (이용실, 김학진, 2010; Van den Bos et. al, 2009).

**9. 옥시토신의 특성 및 비강내 투여의 안전성**

**A. 옥시토신 특성 및 안전성**

- 약리작용 및 효능효과 (점적주사제의 경우)

임신한 자궁의 평활근에 작용하여 자궁의 수축을 항진시킨다. 허가된 효능 효과는 다음과 같다: 다음 경우의 자궁수축의 유발, 촉진 및 자궁출혈의 치료 : 분만유도, 진통미약, 분만후 출혈, 이완성 자궁출혈, 자궁퇴축부전, 제왕절개술(태아만출후), 유산, 인공임신중절.

- 옥시토신 주사제 (KFDA 허가사항)
  - 독성

급성독성 (LD_50_, 단위/kg)

|  | | 정주 | 피하 | 경구 |
| --- | --- | --- | --- | --- |
| 마우스 | 수 | 5140 | >113000 | >113000 |
|  | 암 | 7540 | >113000 | >113000 |
| 랫트 | 수 | 500 | >4510 | >4510 |
|  | 암 | 655 | >4510 | >4510 |

- - 부작용

1) 쇽 : 드물게 쇽이 나타날 수 있으므로 관찰을 충분히 하고 청색증, 허탈 등의 이상이 나타날 경우에는 투여를 중지하고 적절한 처치를 하십시오.

2) 자궁 : 자궁의 과도한 진통, 자궁파열, 경관열상, 양수색전증, 진통미약, 이완출혈 등이 나타날 수 있습니다.

3) 태아, 신생아 : 태아가사가 나타날 수 있으므로 관찰을 충분히 하십시오, 신생아 황달의 발생 빈도가 높다는 보고가 있습니다.

4) 순환기계 : 부정맥, 정맥내 주사 후 홍조 및 빈맥을 동반한 일시적인 혈압강하, 혈압상승, 전흉부통증, 빈맥 등이 나타날 수 있습니다.

5) 소화기계 : 때때로 구역, 구토, 복통, 식욕부진 등이 나타날 수 있습니다.

6) 과민증: 드물게 발진, 아낙필락시 반응 등이 나타날 수 있습니다.

7) 기타 : 무섬유원혈증, 저나트륨혈증, 수분배출의 감소, 수분중독증상 (두통, 졸음, 의식상실, 대발작, 식욕부진, 구역, 구토, 복통, 혈중전해질 농도저하 등) 이 나타날 수 있습니다.

8) 장기투여 : 드물게 항이뇨효과가 나타날 수 있습니다.

9) 근육주사시 주사부위에 동통, 경결이 나타날 수 있습니다.

**B. 옥시토신 비강 내 투여의 안전성 (Macdonald 등 2011)**

옥시토신은 1950년대에 인체 호르몬 중 가장 먼저 정제화 되었다. 옥시토신은 산모의 자궁수축을 촉진한다고 잘 알려져 있으며 산모의 자궁촉진에 사용되는 옥시토신은 정맥을 통한 점적(IV infusion), 주사(IV volus) 방법으로 사용되고 있다. 그 외 산모의 산후자궁회복을 돕고 수유를 촉진하는 효과가 있으며 여기에는 정맥투여 혹은 비강 내 투여 제재가 시판되고 있다. 이 방법은 neuropeptide가 비강 내 처치를 한 후에 혈뇌 장벽을 잘 통과하며 인체에 안전하고 부작용이 없다는 데 근거한다(Born et al., 2002). 최근 옥시토신이 뇌에 작용하여 인간에서 사회성 행동을 증진시키는 역할이 있음이 알려지고 있다. 이는 동물에서 측정할 수 없는 고차원적 정서기능이라는 특성상 옥시토신 비강 내 1회성 투여를 통한 연구들이 시행되어 왔다. 1990년부터 2010년까지 옥시토신 비강 내 투여방법을 이용해 전세계에서 수행된 전 연구를 분석하여 안전성을 평가하였다. 대상은 총38개의 연구로 1529명이 참가하였다. 분석결과 실험실 환경 하에서 18-40 IU 의 단기사용은 부작용과 관련 없는 안전한 것으로 입증되었다. 15세 미만에서의 투여 용량은 18IU 에서 안전성이 입증되었다 (Guastella 등 2011).

**10. 연구실행 계획표**

**A. 연구설계**

본 연구는 이중 맹검, 피험자 내 위약 통제 설계 (cross-over)이다.

**B. 실험절차**

- 본 실험은 환자가 연구 참여에 대해 문서화 된 동의서에 서명을 한 후 연구 절차를 시작할 것이다. 참가자들은 언제든지 연구 참여를 그만둘 수 있다고 알려줄 것이다. 모든 참가자들은 섭식장애 증상의 정도를 평가 받고, 키와 체중을 특정할 것이다. 이전 지능 검사결과가 없는 참가자들은 한국판 지능검사(K-WAIS)를 받을 것이다. 참가자들은 연구자와 함께 DSM-Ⅳ의 평가 준거에 의거해서 진단을 명확하게 하기 위한 간단한 면담을 시행할 것이다. 옥시토신과 플라시보는 비강 내 투여 방법으로 1주일 간격으로 신경심리 검사를 받기 45분 전에 실시한다. 참가자들이 시약을 투여 받는 날에는 알콜과 카페인 섭취를 금하고, 시약을 투여 받기 2시간 전에는 음식과 음료(물 제외) 섭취를 하지 않도록 한다. 본 연구에 참여하는 자는 섭식장애에 대한 영양 및 심리치료를 지속할 것이다.

- 본 연구에서 투여할 옥시토신의 양은 총 40 IU (10 IU, 4번 흡입) 로서 1회 투여 받게 될 것이다 (단 12-15세의 경우 18-24IU 범위에서 적합한 용량 선택). 옥시토신 및 위약 nebulizer 는 1회 사용 후 폐기할 것이다. 옥시토신의 비강 내 투여 시험절차는 다음과 같다. 실험의 첫 단계(Day 1)에서 참가자들은 옥시토신 과 위약 중 하나를 비강 스프레이를 이용해 자가 투여할 것이다. 스프레이는 참가자들에게 45초 간격으로 분사하도록 한다. 회기(옥시토신, 위약) 순서는 피험자와 연구자 모두 맹검 상태에서 진행될 것이다. 그룹할당은 컴퓨터프로그램으로 수행할 것이며 이중맹검 무선할당될 것이다. 옥시토신의 반감기는 5분으로 이는 투여 5분 후 효과가 나타남을 의미하며, 효과는 4시간이내 소멸된다. 위약은 sodium acetate tri-hydrate, acetic acid, chlorobutanol, ethanol, 증류수로 조성되어 있다. 2단계(Day 2)에서는 1단계에서 투여 받은 시약과 다른 스프레이를 투여할 것이다. 본 연구는 cross-over 설계이다. 연구자는 참가자에게 약 30분의 대기시간 동안 설문지 (EDE-Q, DASS 등)를 작성하게 한다. 신경심리검사는 스프레이를 사용한 45분 후에 시작할 것이며, 음식에 대한 주의 편향과 사회 정서 기능을 포함한 변화를 측정할 것이다. 신경심리검사를 마친 후 참가자들은 음료수를 최대한 마시게 하여 섭취량을 기록한다. 첫 날의 실험을 마친 후 참가자에게 24시간 동안의 식사일기를 작성하게 한다.

| **연구 일정표** | | | | | | | |
| --- | --- | --- | --- | --- | --- | --- | --- |
| **방문**  **회수** | **피험자**  **동의** | **설문지**  **작성** | **옥시토신**  **/위약** | **신경심리검사** | **음료수** | **식사일기 1일** | **체중측정** |
| **1회** | X | X | X | X | X | X | X |
| **2회** |  | X | X | X | X | X | X |

**11. 통계처리방법**

A. 신경인지검사(Dot-probe task)에 대한 자료 분석

옳은 반응(correct responses)에 대한 반응시간(reaction time; RT)에 기초할 것이다. RT 가 200 ms 미만이거나 2,000 ms 이상인 경우는 제외할 것임. 편향점수 (bias score)는 다음과 같이 계산한다. [편향점수 = RT: 표적그림(target)과 단서(probe) 가 반대 방향; 편향점수 = (-) RT: 표적그림과 단서가 같은 방향; +: 단서가 섭식장애 관련 표적그림과 같은 방향일 때 단서에 더 빨리 반응한 경우; -: 단서가 섭식장애 관련 표적그림과 같은 방향일 때 단서에 더 느리게 반응한 경우]. 3가지 다른 자극 (즉, 긍정, 부정, 중립) 및 2가지 단서의 위치(표적그림과 같은 방향 혹은 반대 방향)에 대해 repeated measures ANOVAs 를 시행 (이후 paired t-tests)한다.

B. 얼굴표정인식(Facial expression morphing) 과제에 대한 자료분석

옥시토신과 위약 간에 정서유형에 따라 정서인식의 민감도와 정확성에 차이가 있는지 확인하기 위해, 옥시토신과 위약의 집단구분을 집단 간 요인으로, 정서유형을 집단 내 요인으로 하여 2요인 반복측정 변량분석을 실시한다. 이를 통해 정서유형의 주효과와 집단간 주효과, 정서유형과 집단 간의 상호작용효과를 확인할 것이며, 정서유형의 주효과가 유의미할 경우 사후검증을 통해 정서유형 별로 어떤 차이가 있는지 탐색할 것이다.

C. 옥시토신과 위약 간 비교 분석

Repeated measures ANOVAs 를 시행할 것이다. 본 연구에서는 그룹(옥시토신, 위약)과 표적그림(target) 간 상호작용이 있을 것이라는 가설 하에 옥시토신이 긍정자극에 대한 반응을 향상시킬 것으로 예상한다.

**연구 2. 유전연구**

질환의 특성과 옥시토신 수용체 유전자 다형성과의 연관을 규명하기 위해, 피험자의 혈액 (약 3cc) 및 구강상피 세포를 채취한다. 대상 유전자는 dbSNP에 등록된 OXTR 유전자의 SNP 중에서, 이 질환과의 관련을 보이는 SNP 들이다.

1. **대상**

채혈에 동의한 섭식장애 환자 50명 및 부모 100명

대조군으로써 신경증 환자 50명 및 건강한 정상인 50명

1. **채혈 및 유전학적 분석**

OXTR promoter 부위의 methylation 상태 분석;

섭식장애 병리와 OXTR 유전자의 epigenetic silencing과의 관련성을 연구하기 위해, OXTR 유전자의 프로모터 부위의 methylation 상태를 bisulfite sequencing을 이용하여 분석한다. 혈액에서 정제한 DNA의 bisulfate 전환은 methylSEQr Bisulfite Conversion Kit (Applied Biosystems, CA, USA)를 이용한다. MT2를 포함하는 OTR 유전자의 프로모터 부위를 bisulfite를 처리한 DNA와 처리하지 않은 DNA를 주형으로 하여 PCR을 이용하여 증폭한다. PCR에 이용할 primer의 염기서열은 다음과 같다.

5’-AAGTTTTGGAATTTTTGATTTG-3’

5’-CCAATAAAAAACCTCAACTTAAC-3’.

PCR 조건은 95°C, 3분 1 회, 95°C, 1분, 56.8°C, 1분, 72°C, 3분 55회, 72°C, 5분 1회로 수행한다. 이렇게 얻은 PCR 산물은 agarose gel 전기영동하여 분리하고, Gel Extraction Kit (Qiagen, CA, USA)를 이용하여 정제한다. 정제된 PCR 산물은 TOPO TA vector (Invitrogen, CA, USA)에 클론닝한다. 각 sample로부터 10개의 colony를 취하여 염기서열을 결정하여, 각 CpG 자리의 methylation 정도를 백분율로 계산한다.

d. 유전학적 통계분석

DNA methylation 정도와 질환특성 등과의 상관관계는 Pearson correlation analysis 를 이용할 것이다. 섭식장애와 Oxytocin 유전자형 간의 관련성 및 성격 특성과의 관련성은 χ2 test 및 MANOVA 를 이용하여 검증할 것이다. 유전자 다형성의 빈도분석을 위해서는 Kruskal-Wallis analysis 를 시행할 것이다.

**12. 피해에 대한 보상**

- 옥시토신의 비강 내 투여 방법은 인체에 무해하다고 알려져 있다. 옥시토신 투여로 인한 직접적인 부작용들은 매우 드물지만 쇽, 과민증 등이 있다. 이러한 증상들은 본 연구책임자가 즉각적으로 의학적 도움을 구하여 처치할 것이며 이러한 부작용을 응급처치하는 데 발생되는 직접적인 의료비용은 연구자가 부담할 것이다. 그 외의 추가적인 보상은 없을 것이다. 다른 위험이 발생할 경우 의료진은 즉각 그 사실을 주지하여 서울백병원 임상시험심사위원회에 알릴 것이다.

**13. 자료관리 및 개인정보보안**

본 연구의 결과에 개인 신상정보는 포함되지 않으며 이를 위하여 개개 환자 별 코드 변호를 부여하여 관리함으로써 연구결과의 출판 및 연구 결과가 공개될 때에도 개인 신상정보는 포함되지 않도록 할 것이다. 또 연구책임자는 코드화 되기 전 수집된 개인 정보들이 누출되지 않도록 주의 감독에 대한 책임을 질 것이다.

**14. 참고문헌**

**References**

Barrett, J. C., Fry, B., Maller, J. and Daly, M. J. (2005) Haploview: analysis and visualization of LD and haplotype maps. *Bioinformatics* **21,** 263-265.

Bartz, J. A. and Hollander, E. (2008) Oxytocin and experimental therapeutics in autism spectrum disorders. In: *Advances in Vasopressin and Oxytocin: From Genes to Behaviour to Disease*. pp. 451-462. Eds. I. D. Neumann, R. Landgraf.

Baumgartner, T., Heinrichs, M., Vonlanthen, A., Fischbacher, U. and Fehr, E. (2008) Oxytocin shapes the neural circuitry of trust and trust adaptation in humans. *Neuron* **58,** 639-650.

Born, J., Lange, T., Kern, W., McGregor, G. P., Bickel, U. and Fehm, H. L. (2002) Sniffing neuropeptides: a transnasal approach to the human brain. *Nature Neuroscience* **5,** 514-516.

Brooks (2010) An investigation of the neural processes associated with eating disorder symptomatology. University of London: London.

Brooks, S., Prince, A., Stahl, D., Campbell, I. C. and Treasure, J. (2011) A systematic review and meta-analysis of cognitive bias to food stimuli in people with disordered eating behaviour. *Clinical Psychology Review* **31,** 37-51.

Brown TA, Chorpita BF, Korotitsch W and DH., B. (1997) Psychometric properties of the Depression Anxiety Stress Scales (DASS) in clinical samples. *Behaviour Research and Therapy* **35,** 79-89.

Campbell, I. C., Mill, J., Uher, R. and Schmidt, U. (2011) Eating disorders, gene-environment interactions and epigenetics. *Neuroscience and Biobehavioral Reviews* **35,** 784-793.

Cardi V and Treasure J (2011) Social reward and rejection sensitivity in Eating Disorders: an investigation of attentional bias and early experiences. *Biological Psychiatry*.

Cserjesi R, Vermeulen N, Lenard L and O., L. (2011 in press) Reduced capacity in automatic processing of facial expression in restrictive anorexia nervosa and obesity. *Psychiatry Research*.

Davies H, S. U., Stahl D, Tchanturia K. (2011 in press) Evoked facial emotional expression and emotional experience in people with anorexia nervosa. *International Journal of Eating Disorders*.

Demitrack, M. A., Lesem, M. D., Listwak, S. J., Brandt, H. A., Jimerson, D. C. and Gold, P. W. (1990) CSF oxytocin in anorexia nervosa and bulimia nervosa -clinical and pathophysiologic considerations *American Journal of Psychiatry* **147,** 882-886.

Domes, G., Heinrichs, M., Glascher, J., Buchel, C., Braus, D. F. and Herpertz, S. C. (2007a) Oxytocin attenuates amygdala responses to emotional faces regardless of valence. *Biological Psychiatry* **62,** 1187-1190.

Domes, G., Heinrichs, M., Michel, A., Berger, C. and Herpertz, S. C. (2007b) Oxytocin improves "mind-reading" in humans. *Biological Psychiatry* **61,** 731-733.

Fairburn, C. G. and Beglin, S. J. (1994) Assessment of eating disorders - interview or self-report questionnaire. *International Journal of Eating Disorders* **16,** 363-370.

Fairburn, C. G. and Cooper, Z. (1993) *The Eating Disorders Examination (12th edition). Binge eating: Nature, assessment and treatment.* Guilford: New York, NY.

Friederich, H. C., Kumari, V., Uher, R., Riga, M., Schmidt, U., Campbell, I. C., Herzog, W. and Treasure, J. (2006) Differential motivational responses to food and pleasurable cues in anorexia and bulimia nervosa: a startle reflex paradigm. *Psychological Medicine* **36,** 1327-1335.

Giel, K. E., Teufel, M., Friederich, H.-C., Hautzinger, M., Enck, P. and Zipfel, S. (2011) Processing of pictorial food stimuli in patients with eating disorders—A systematic review. *International Journal of Eating Disorders***,** Epub ahead of print, DOI: 10.1002/eat.20785.

Gillberg, I. C., Billstedt, E., Wentz, E., Anckarsater, H., Rastam, M. and Gillberg, C. Attention, executive functions, and mentalizing in anorexia nervosa eighteen years after onset of eating disorder. *Journal of Clinical and Experimental Neuropsychology* **32,** 358-365.

Gimpl, G. and Fahrenholz, F. (2001) The Oxytocin Receptor System: Structure, function, and regulation. *Physiological Reviews* **81,** 629-683.

Gotlib, I. H., Krasnoperova, E., Yue, D. N. and Joormann, J. (2004) Attentional biases for negative interpersonal stimuli in clinical depression. *Journal of Abnormal Psychology* **113,** 127-135.

Gregory, S. G., Connelly, J. J., Towers, A. J., Johnson, J., Biscocho, D., Markunas, C. A., Lintas, C., Abramson, R. K., Wright, H. H., Ellis, P., Langford, C. F., Worley, G., Delong, G. R., Murphy, S. K., Cuccaro, M. L., Persico, A. and Pericak-Vance, M. A. (2009) Genomic and epigenetic evidence for oxytocin receptor deficiency in autism. *BMC Medicine* **7**.

Guastella, A. J., Einfeld, S. L., Gray, K. M., Rinehart, N. J., Tonge, B. J., Lambert, T. J. and Hickie, I. B. (2010) Intranasal Oxytocin Improves Emotion Recognition for Youth with Autism Spectrum Disorders. *Biological Psychiatry* **67,** 692-694.

Harris, E. C. and Barraclough, B. (1998) Excess mortality of mental disorder. *British Journal of Psychiatry* **173,** 11-53.

Harrison, A., Tchanturia, K. and Treasure, J. (2010) Attentional Bias, Emotion Recognition, and Emotion Regulation in Anorexia: State or Trait? *Biological Psychiatry* **68,** 755-761.

Heinrichs, M., Baumgartner, T., Kirschbaum, C. and Ehlert, U. (2003) Social support and oxytocin interact to suppress cortisol and subjective responses to psychosocial stress. *Biological Psychiatry* **54,** 1389-1398.

Hjern, A., Lindberg, L. and Lindblad, F. (2006) Outcome and prognostic factors for adolescent female in-patients with anorexia nervosa: 9-to 14-year follow-up. *British Journal of Psychiatry* **189,** 428-432.

Hudson, J. I., Hiripi, E., Pope, H. G. and Kessler, R. C. (2007) The prevalence and correlates of eating disorders in the national comorbidity survey replication. *Biological Psychiatry* **61,** 348-358.

Inoue, T., Kimura, T., Azuma, C., Inazawa, J., Takemura, M., Kikuchi, T., Kubota, Y., Ogita, K. and Saji, F. (1994) Structural organization of the human oxytocin receptor gene. *Journal of Biological Chemistry* **269,** 32451-32456.

Jiang, T., Soussignan, R., Rigaud, D. and Schaal, B. (2010) Pleasure for visual and olfactory stimuli evoking energy-dense foods is decreased in anorexia nervosa. *Psychiatry Research* **180,** 42-47.

Kim, Y. R., Lim, S. J. and Treasure, J. (2011) Different Patterns of Emotional Eating and Visuospatial Deficits Whereas Shared Risk Factors Related with Social Support between Anorexia Nervosa and Bulimia Nervosa. *Psychiatry Investigation* **8,** 9-14.

Kirsch, P., Esslinger, C., Chen, Q., Mier, D., Lis, S., Siddhanti, S., Gruppe, H., Mattay, V. S., Gallhofer, B. and Meyer-Lindenberg, A. (2005) Oxytocin modulates neural circuitry for social cognition and fear in humans. *Journal of Neuroscience* **25,** 11489-11493.

Kosfeld, M., Heinrichs, M., Zak, P. J., Fischbacher, U. and Fehr, E. (2005) Oxytocin increases trust in humans. *Nature* **435,** 673-676.

Kusui, C., Kimura, T., Ogita, K., Nakamura, H., Matsumura, Y., Koyama, M., Azuma, C. and Murata, Y. (2001) DNA methylation of the human oxytocin receptor gene promoter regulates tissue-specific gene suppression. *Biochemical and Biophysical Research Communications* **289,** 681-686.

Lerer, E., Levi, S., Salomon, S., Darvasi, A., Yirmiya, N. and Ebstein, R. P. (2008) Association between the oxytocin receptor (OXTR) gene and autism: relationship to Vineland Adaptive Behavior Scales and cognition. *Molecular Psychiatry* **13,** 980-988.

Lopez, C., Tchanturia, K., Stahl, D. and Treasure, J. (2008) Central coherence in eating disorders: a systematic review. *Psychological Medicine* **38,** 1393-1404.

Lovibond, S. H. and Lovibond, P. F. (1993 ) *Manual for the Depression Anxiety Stress Scales (DASS)*. Psychology Foundation: Sydney.

Macleod, C., Mathews, A. and Tata, P. (1986) Attentional bias in emotional disorders. *Journal of Abnormal Psychology* **95,** 15-20.

Mansell, W., Clark, D. M., Ehlers, A. and Chen, Y. P. (1999) Social anxiety and attention away from emotional faces. *Cognition & Emotion* **13,** 673-690.

Mogg, K. and Bradley, B. P. (1998) A cognitive-motivational analysis of anxiety. *Behaviour Research and Therapy* **36,** 809-848.

Odent, M. (2010) Autism and anorexia nervosa: Two facets of the same disease? *Medical Hypotheses* **75,** 79-81.

Oldershaw, A., Hambrook, D., Stahl, D., Tchanturia, K., Treasure, J. and Schmidt, U. (2011) The socio-emotional processing stream in Anorexia Nervosa. *Neuroscience and Biobehavioral Reviews* **35,** 970-988.

Rabinowitz, D. and Laird, N. (2000) A unified approach to adjusting association tests for population admixture with arbitrary pedigree structure and arbitrary missing marker information. *Human Heredity* **50,** 211-223.

Sham, P. C. and Curtis, D. (1995) An extended transmission/disequilibrium test (TDT) for multiallele marker loci. *Annals of Human Genetics* **59,** 323-336.

Soussignan, R., Jiang, T., Rigaud, D., Royet, J. P. and Schaal, B. (2010) Subliminal fear priming potentiates negative facial reactions to food pictures in women with anorexia nervosa. *Psychological Medicine* **40,** 503-514.

Uher, R., Murphy, T., Brammer, M. J., Dalgleish, T., Phillips, M. L., Ng, V. W., Andrew, C. M., Williams, S. C. R., Campbell, I. C. and Treasure, J. (2004) Medial prefrontal cortex activity associated with symptom provocation in eating disorders. *American Journal of Psychiatry* **161,** 1238-1246.

Vos, T., Mathers, C., Herrman, H., Harvey, C., Gureje, O., Bui, D., Watson, N. and Begg, S. (2001) The burden of mental disorders in Victoria, 1996. *Social Psychiatry and Psychiatric Epidemiology* **36,** 53-62.

Wagner, A., Aizenstein, H., Mazurkewicz, L., Fudge, J., Frank, G. K., Putnam, K., Bailer, U. F., Fischer, L. and Kaye, W. H. (2008) Altered insula response to taste stimuli in individuals recovered from restricting-type anorexia nervosa. *Neuropsychopharmacology* **33,** 513-523.

Watson, D., Clark, L. A. and Tellegen, A. (1988) Development and validation of brief measures of positive and negative affect - the PANAS scales *Journal of Personality and Social Psychology* **54,** 1063-1070.

Wentz, E., Gillberg, I. C., Anckarsater, H., Gillberg, C. and Rastam, M. (2009) Adolescent-onset anorexia nervosa - missing half of the story? Reply. *British Journal of Psychiatry* **194,** 565-565.

Wu, S. P., Jia, M. X., Ruan, Y., Liu, J., Guo, Y. Q., Shuang, M., Gong, X. H., Zhang, Y. B., Yang, X. L. and Zhang, D. (2005) Positive association of the oxytocin receptor gene (OXTR) with autism in the Chinese Han population. *Biological Psychiatry* **58,** 74-77.

Yum, T. H., Park, Y. S., Oh, K. J., Kim, J. G. and Lee, H. Y. (1992) *The manual of Korean-Wechsler adult intelligence scale*. Korean Guidance Press: Seoul.

Zucker, N. L., Losh, M., Bulik, C. M., Labar, K. S., Piven, J. and Pelphrey, K. A. (2007) Anorexia nervosa and autism spectrum disorders: Guided investigation of social cognitive endophenotypes. *Psychological Bulletin* **133,** 976-1006.

양재원(2009). 사회불안증상과 얼굴표정의 정서인식 편향. 연세대학교 박사학위 청구 논문.

이용실, 김학진 (2010). 신뢰로운 얼굴이 투자 결정에 미치는 영향: 눈, 코, 입의 영향비교. 한국심리학회지 사회 및 성격 24, 117-131.
